# Acute and Chronic Effects of Accentuated Eccentric Loading vs. Constant-Load Resistance Training: A Systematic Review and Meta-analysis

**DOI:** 10.1007/s40279-026-02422-7

**Published:** 2026-04-07

**Authors:** Xing Zhang, Jonathon Weakley, Hansen Li, Daniel Marcos-Frutos, Amador García-Ramos

**Affiliations:** 1 Department of Physical Education and Sports, Faculty of Sport Sciences, University of Granada, Granada, Spain; 2 School of Behavioural and Health Sciences, Australian Catholic University, Brisbane, QLD Australia; 3 Sports Performance, Recovery, Injury and New Technologies (SPRINT) Research Centre, Australian Catholic University, Brisbane, QLD Australia; 4 Carnegie Applied Rugby Research (CARR) Centre, Carnegie School of Sport, Leeds Beckett University, Leeds, UK; 5 School of Physical Education, Sichuan Agricultural University, Ya’an, China; 6 Department of Sports Sciences and Physical Conditioning, Faculty of Education, Universidad Católica de La Santísima Concepción, Concepción, Chile

## Abstract

**Background:**

Accentuated eccentric loading (AEL) is a resistance training (RT) method applying greater eccentric- than concentric-phase load to intensify the training stimulus; however, despite its common use, a comprehensive and quantitative review remains lacking.

**Objective:**

The aim was to compare acute responses and chronic adaptations between AEL and constant-load RT, and examine whether effects vary by AEL protocol (submaximal, maximal, supramaximal).

**Methods:**

PubMed, Web of Science, Embase, and EBSCO were searched from inception through July 3, 2024; eligible English-language studies were included. Pooled and subgroup meta-analyses were performed using random-effects models.

**Results:**

Forty-nine studies involving 773 participants were included. Although considerable variance exists in certain outcomes, our estimated effects suggest that, compared to constant-load RT, AEL results in (1) similar acute responses in loads lifted during the concentric phase (standardized mean difference [SMD] =  − 0.16; *p* = 0.48), mechanical performance at submaximal loads during the concentric phase (SMD =  − 0.07; *p* = 0.37), countermovement jump height both immediately (SMD =  − 0.06; *p* = 0.86) and delayed (SMD =  − 0.23; *p* = 0.44) post-intervention, maximal voluntary isometric force immediately post-intervention (SMD = 0.03; *p* = 0.89), blood lactate concentration during the intervention (SMD =  − 0.06; *p* = 0.78), testosterone concentration immediately post-intervention (SMD = 0.68; *p* = 0.15), creatine kinase concentration both immediately (SMD = 0.09; *p* = 0.72) and delayed (SMD = 0.14; *p* = 0.48) post-intervention, cortisol concentration immediately post-intervention (SMD = 0.39; *p* = 0.05), heart rate during the intervention (SMD = 1.18; *p* = 0.07), acute muscle swelling immediately post-intervention (SMD = 0.26; *p* = 0.42), muscle electrical activity during the concentric phase (SMD =  − 0.01; *p* = 0.90), and muscle soreness both immediately (SMD = 0.28; *p* = 0.30) and delayed (SMD = 0.18; *p* = 0.28) post-intervention; (2) greater acute responses in blood lactate concentration immediately post-intervention (SMD = 0.44; *p* = 0.03), growth hormone concentration immediately post-intervention (SMD = 0.50; *p* = 0.01), muscle electrical activity during the eccentric phase (SMD = 0.37; *p* = 0.01), and rating of perceived exertion immediately post-intervention (SMD = 1.72; *p* = 0.01); (3) similar chronic adaptations in maximal concentric strength (SMD = 0.12; *p* = 0.41), maximal eccentric strength (SMD = 0.19; *p* = 0.58), maximal isometric strength (SMD = 0.03; *p* = 0.93), countermovement jump height (SMD = 0.04; *p* = 0.87), muscle fascicle angle (SMD =  − 0.10; *p* = 0.77), muscle fascicle length (SMD = 0.90; *p* = 0.17), and muscle cross-sectional area (SMD =  − 0.06; *p* = 0.84).

**Conclusion:**

While AEL augments the eccentric-phase stimulus (higher eccentric load and muscle electrical activity), it also increases metabolic stress and perceived effort, implying a need for longer, more frequent inter-set rests and longer between-session recovery. Given the lack of evidence for superior chronic benefits in strength or muscle architecture over constant-load RT, practitioners should consider these factors carefully.

**Protocol Registration:**

The original protocol for this review was prospectively registered with the International Prospective Register of Systematic Reviews (PROSPERO) in July 2024 (CRD42024561673).

**Supplementary Information:**

The online version contains supplementary material available at 10.1007/s40279-026-02422-7.

## Key Points

**Table:** 

Accentuated eccentric loading enhances muscle electrical activity during the eccentric phase compared to constant-load resistance training.
Accentuated eccentric loading causes higher perceived exertion and increased metabolic stress compared to constant-load resistance training.
Accentuated eccentric loading does not offer superior benefits in strength-related or muscle architectural adaptations compared to constant-load resistance training.

## Introduction

Resistance training (RT) offers a wide range of functional and health-related benefits, such as increasing maximal strength [1, 2], improving jumping ability [3, 4], enhancing sprint performance [5], and improving conditions like cardiovascular disease [6], type 2 diabetes [7], and osteoporosis [8]. However, to achieve these benefits, it is essential to carefully plan and regulate various training variables, including load, volume, frequency, inter-set rest, and set structure [9–11]. Among these variables, load plays a crucial role in determining the effectiveness of RT, especially when the goal is to maximize maximal force capacity (e.g., one repetition maximum [1RM]) [2, 12].

Traditional RT typically involves performing a series of repetitions with a constant load, where the load remains the same during both the concentric and eccentric phases of the exercise [13]. However, muscles are capable of producing up to 50% more force during maximum eccentric contractions compared to concentric contractions [14]. This discrepancy can be attributed to differences in the muscle’s mechanical properties (e.g., prolonged cross-bridge attachment and enhanced elastic energy utilization) and neuromuscular control mechanisms (e.g., altered neural activation patterns and distinct motor unit recruitment strategies) [15, 16]. As a result, using constant loading may provide a submaximal stimulus during the eccentric phase, leading to lower motor unit recruitment and firing rates, reduced sarcoplasmic calcium release, and potentially a diminished stimulus for myocellular adaptation [17]. This, in turn, can hinder the development of muscle strength and hypertrophy [18]. Therefore, it is logical for strength and conditioning experts to seek alternative training methods that apply overload during the eccentric phase to provide a more demanding training stimulus [14].

Accentuated eccentric loading (AEL) involves using a load that is greater during the eccentric portion of the movement compared to the concentric portion [14]. For example, AEL can be observed when performing RT with weight releasers. Weight releasers attach to the barbell using hooks and stay in place during the eccentric (lowering) phase of the first repetition of an exercise [19]. At the bottom of the movement, when the weight releasers touch the floor, they automatically detach, leaving only the original barbell weight for the concentric (lifting) phase [19]. Additionally, other methods such as computer-driven systems [20], pneumatic assistance [21], and manual adjustments of weight during the eccentric and concentric phases can also be used to achieve AEL in RT [22]. Previous studies have confirmed that AEL provides a greater training stimulus during the eccentric phase than constant-load RT [23–25], while also eliciting a within-set potentiation effect that enhances mechanical performance during the concentric phase [26, 27]. Given this, AEL is considered to have the potential to induce superior training adaptations [19, 28, 29].

AEL can be categorized into three categories based on the relative load, expressed as a percentage of the 1RM in the concentric phase, applied during the eccentric phase [14, 30]: (1) *submaximal AEL:* the eccentric load is less than the individual’s 1RM (e.g., concentric 60% 1RM with eccentric 80% 1RM); (2) *maximal AEL:* the eccentric load is equal to the individual’s 1RM (e.g., concentric 60% 1RM with eccentric 100% 1RM); and (3) *supramaximal AEL:* the eccentric load exceeds the individual’s 1RM (e.g., concentric 60% 1RM with eccentric 120% 1RM). While these AEL methods all involve an eccentric load that is heavier than the concentric load, they differ in the magnitude of the eccentric load relative to the 1RM, which can lead to varying outcomes [2, 31, 32].

To date, numerous experimental studies have been conducted to explore the differences between AEL and constant-load RT [19, 28, 29, 33–37]. However, these studies have not reached a unified conclusion and, at times, have even produced contradictory results. Although Wagle et al. [14] conducted a narrative review on this topic, their work primarily provided a descriptive introduction and did not include a quantitative synthesis. Therefore, a comprehensive systematic review and meta-analysis is still needed to clarify the differences between AEL and constant-load RT. The primary aim of the current study is to establish evidence for the effects of AEL on acute mechanical, metabolic, and perceptual variables, as well as chronic training adaptations; and quantify whether differences can be influenced by the specific AEL protocol employed, categorized as submaximal, maximal, or supramaximal AEL.

## Methods

### Registration of Systematic Review and Meta-analysis Protocol

This systematic review and meta-analysis adhered to the guidelines outlined in the *Cochrane Handbook for Systematic Reviews of Interventions*, version 5.1.0, and followed the Preferred Reporting Items for Systematic Reviews and Meta-Analyses (PRISMA) 2020 checklist [38]. The original protocol for this review was prospectively registered with the International Prospective Register of Systematic Reviews (PROSPERO) in July 2024 (CRD42024561673).

### Information Sources and Search Strategy

A comprehensive systematic literature search was conducted using the following English electronic databases: PubMed, Web of Science, Embase, and EBSCO. The search covered the period from the inception of each database to July 3rd, 2024. The following syntax was adapted for each database: "Accentuated Eccentric Load," "Eccentric Overload," "Additional Eccentric Load," "Enhanced Eccentric Load," "Eccentric Focused Training," and "Augmented Eccentric Load." The specific search strategies for each database are detailed in Supplementary 1 (see the electronic supplementary material). Free-text terms were utilized based on preliminary searches to achieve a balance between search sensitivity and precision, deliberately avoiding the use of controlled vocabulary (e.g., Medical Subject Headings). The search strategy focused exclusively on terms related to the intervention and did not incorporate population or subject-specific information. Abstracts, letters to editors, commentaries, conference proceedings, and master and doctoral theses were excluded from this review. To ensure a thorough inclusion of relevant studies, a secondary search was conducted. This process involved using studies that met the inclusion criteria to identify additional potential studies via the "Related articles" feature on Google Scholar. Furthermore, the reference lists of the studies meeting the inclusion criteria were also screened.

### Eligibility Criteria

A PICO framework was applied to establish the eligibility criteria for including or excluding studies, as follows:

- P (Population): Healthy individuals without known medical conditions or injuries.
- I (Intervention): RT with AEL.
- C (Comparison): AEL versus constant-load RT.
- O (Outcomes): Acute effects on mechanical, metabolic, and perceptual variables, as well as chronic effects on strength and structural adaptations.

Only peer-reviewed studies published in English were considered for inclusion [39, 40]. For chronic studies, only interventions lasting at least 4 weeks were considered [41]. In this study, AEL was defined as RT exercises involving both eccentric and concentric actions, where the eccentric load exceeds the concentric load. In contrast, constant-load RT uses equivalent loads during both the eccentric and concentric phases. Plyometric training was excluded due to differences in principles, objectives, and implementation compared to typical RT [42]. Additionally, some studies involving jump training with AEL utilize a control group that only performs bodyweight exercises, which does not meet the comparison criteria required for this review. Similarly, flywheel training was excluded because it typically lacks a load-matched RT control group. Furthermore, whether eccentric overloading occurs during flywheel training depends on factors such as the force applied during the concentric phase, the movement technique during the eccentric phase, and the settings of the flywheel equipment [43, 44].

### Study Selection

All records were initially downloaded into EndNote X8 (Clarivate Analytics, Philadelphia, PA, USA), where duplicates were removed based on author, title, and publication year. Subsequently, titles and abstracts were screened to determine initial eligibility. For those records that passed this stage, full texts were retrieved and further assessed. The eligibility assessment was conducted independently by two reviewers (XZ and HSL). Any discrepancies during the selection process were resolved through discussion between the two reviewers, with a third reviewer brought in if necessary (AGR).

### Data Items and Data Extraction Process

For studies that met the inclusion criteria, the following data were extracted into an Excel spreadsheet: (1) study identification information, (2) study design, (3) sample size, (4) participant details, (5) training prescription, and (6) means and standard deviations (SDs) for relevant outcome measures. When data were reported exclusively in figures, the GetData Graph Digitizer 2.26 software (GetData Software Pty Ltd, Kogarah, NSW, Australia) was used to extract the information. The data extraction was conducted independently by two reviewers (XZ and HSL). Any discrepancies were resolved through discussion between the reviewers, with a third reviewer consulted if necessary (AGR).

### Assessment of Bias and Evidence Quality

A risk of bias assessment was conducted using a modified version of the Cochrane Collaboration's tool for evaluating bias in eligible studies [45]. The modifications included removing the performance bias item and adding items for outcome assessment bias, effort bias, and familiarization bias. The performance bias item was removed because blinding participants and personnel in supervised exercise intervention studies was considered impractical [46–48]. Outcome assessment bias was particularly important in this review, emphasizing the need for reliable and appropriate equipment or instruments to evaluate outcomes in each study [49]. A study was considered to have a low risk of outcome assessment bias if previous research had validated the reliability of the tools used to measure the relevant variables, or if the study itself showed at least an acceptable reliability. Effort bias was also a crucial factor, as variations in participant effort could affect these measurements [49]. A study was judged to have a low risk of effort bias if the authors clearly stated that all participants performed the lifting phase of the repetitions with maximal effort. Familiarization bias was another key consideration, as participants’ familiarity with the study protocols could influence their training performance [49]. A study was considered to have a low risk of familiarization bias if the authors specified that a familiarization session was conducted or if the participants had RT experience and were accustomed to the exercises used in the study. The risk of bias assessment was based solely on the information reported in the published papers, without additional input from the study authors. This assessment was independently conducted by two reviewers (XZ and DMF), with any discrepancies resolved through discussion. If needed, a third reviewer was consulted (AGR).

The quality of evidence was assessed using the Grading of Recommendations Assessment, Development, and Evaluation (GRADE) system. Initially, the evidence was rated as high quality, but it could be downgraded by one level to moderate, low, or very low based on the following factors: (1) *risk of bias:* if ≥ 50% of the studies in a meta-analysis had one or more items flagged as high risk of bias; (2) *imprecision:* if the total sample size in a meta-analysis was fewer than 100 participants; (3) *inconsistency:* if there was high statistical heterogeneity within a meta-analysis; and (4) *indirectness:* if the study did not directly compare AEL with constant-load RT in the same target population.

### Preprocessing of Variables

Based on the timing of acute variable measurements, we categorized the acute impacts as "during" (measured during the intervention), "immediate" (within 1 h post-intervention), and "delayed" (1–7 days post-intervention) effects. In some cases, a variable was reported multiple times within a study. To minimize bias from duplicate inclusions, only the data representing the maximum average between AEL and constant-load RT were used. Take, for example, a study measuring blood lactate levels after each set of an intervention. In set 1, the lactate concentration was 10 mmol/L for AEL and 11 mmol/L for constant-load RT, resulting in an average of 10.5 mmol/L. In set 2, the lactate concentrations were 8 mmol/L for AEL and 14 mmol/L for constant-load RT, with an average of 11 mmol/L. In this case, we selected the data from set 2 because they represented the maximum average between AEL and constant-load RT. Data from set 1 were excluded to prevent duplicate inclusion of the same variable across the analysis.

Some included studies may have assessed concentric mechanical performance at submaximal loads using multiple variables (e.g., mean/peak velocity, mean/peak power, mean/peak force), and different studies may have reported different variables. To avoid double-counting, we used one proxy per study selected via a predefined hierarchy: mean velocity → peak velocity → mean power → peak power. This hierarchy was chosen because mean velocity is the preferred metric for non-ballistic resistance exercises, as it better represents performance across the concentric phase. In contrast, peak velocity is typically prioritized in ballistic actions. Given that this study focused on non-ballistic exercises performed with high loads, mean velocity was therefore selected as the most appropriate metric. Moreover, velocity was prioritized over mechanical power to enhance comparability across studies, as for a given relative load (%1RM), velocity is more consistent, whereas power is additionally influenced by the absolute load displaced [50, 51]. Furthermore, velocity can be measured more reliably than power when using linear position transducers. Accordingly, power outcomes were considered only when velocity data were not reported. When a study reported more than one eligible variable, we extracted the highest-ranked available measure and used it in the meta-analysis of concentric mechanical performance at submaximal loads.

### Statistical Analysis

When two or more studies reported the same outcome, a random-effects meta-analysis was conducted using Stata 18 software (StataCorp LLC, Texas, USA). Model parameters were calculated using the maximum likelihood method, with observations weighted by the inverse of the sampling variance. The pooled effect between AEL and constant-load RT was expressed as standardized mean differences (SMDs) with Hedges’ g correction, along with a 95% confidence interval (CI). Additionally, 95% prediction intervals (PIs) were computed to estimate the potential range of effect sizes in future studies, capturing not only the uncertainty of effect sizes within the current studies but also accounting for heterogeneity across studies. For acute indicators, the SMD, 95% CI, and 95% PI were calculated based on the mean and SD. For chronic indicators, these metrics were calculated based on the change in mean and SD from pre- (baseline) to post-intervention. If the change in SD was not provided, it was computed according to the guidelines outlined in the *Cochrane Handbook for Systematic Reviews of Interventions* [52]. Since none of the studies reported correlations between pre- and post-intervention measures, a conservative correlation coefficient of 0.5 was used [53]. The formula is as follows:

$${\mathrm{SD}}_{\mathrm{change}}=\sqrt{({\mathrm{SD}}_{\mathrm{baseline}}^{2}+{\mathrm{SD}}_{\mathrm{final}}^{2}-(2\times 0.5\times {\mathrm{SD}}_{\mathrm{baseline}}\times {\mathrm{SD}}_{\mathrm{final}})).}$$

Additionally, in cases where some studies reported standard error (SE) instead of SD, we used the following formula for conversion:

$$\mathrm{SD}=\mathrm{SE}\times \sqrt{N}.$$

The magnitude of the SMD was interpreted as follows: small (0.20–0.49), moderate (0.50–0.79), and large (> 0.80) [46]. Subgroup analyses compared each AEL subtype—submaximal, maximal, and supramaximal—with constant-load RT. When a study included multiple AEL subtypes, each eligible subtype–control contrast was entered as a separate trial in the meta-analysis. For example, Ojasto and Häkkinen [54] examined three AEL conditions (concentric 70% 1RM with eccentric 80%, 90%, and 100% 1RM) for blood lactate, which were treated as three trials (eccentric overload of 80% and 90% 1RM categorized as submaximal AEL; eccentric overload of 100% 1RM categorized as maximal AEL). Publication bias was assessed using Egger’s test and by inspecting funnel plots when there were ten or more studies available for the same outcome [55]. Heterogeneity among the studies was evaluated using the *I*^2^ statistic, which represents the percentage of total variation in estimated effects across studies due to heterogeneity rather than chance. An *I*^2^ value of 75% or higher was considered indicative of high heterogeneity [56, 57]. Sensitivity analysis was performed by excluding studies that applied different training prescriptions between AEL and constant-load RT, including variations in load, sets, or repetitions per set. In all analyses, *p* values < 0.05 and CIs that did not include zero were considered statistically significant.

## Results

### Studies Selection

**Fig. 1:**
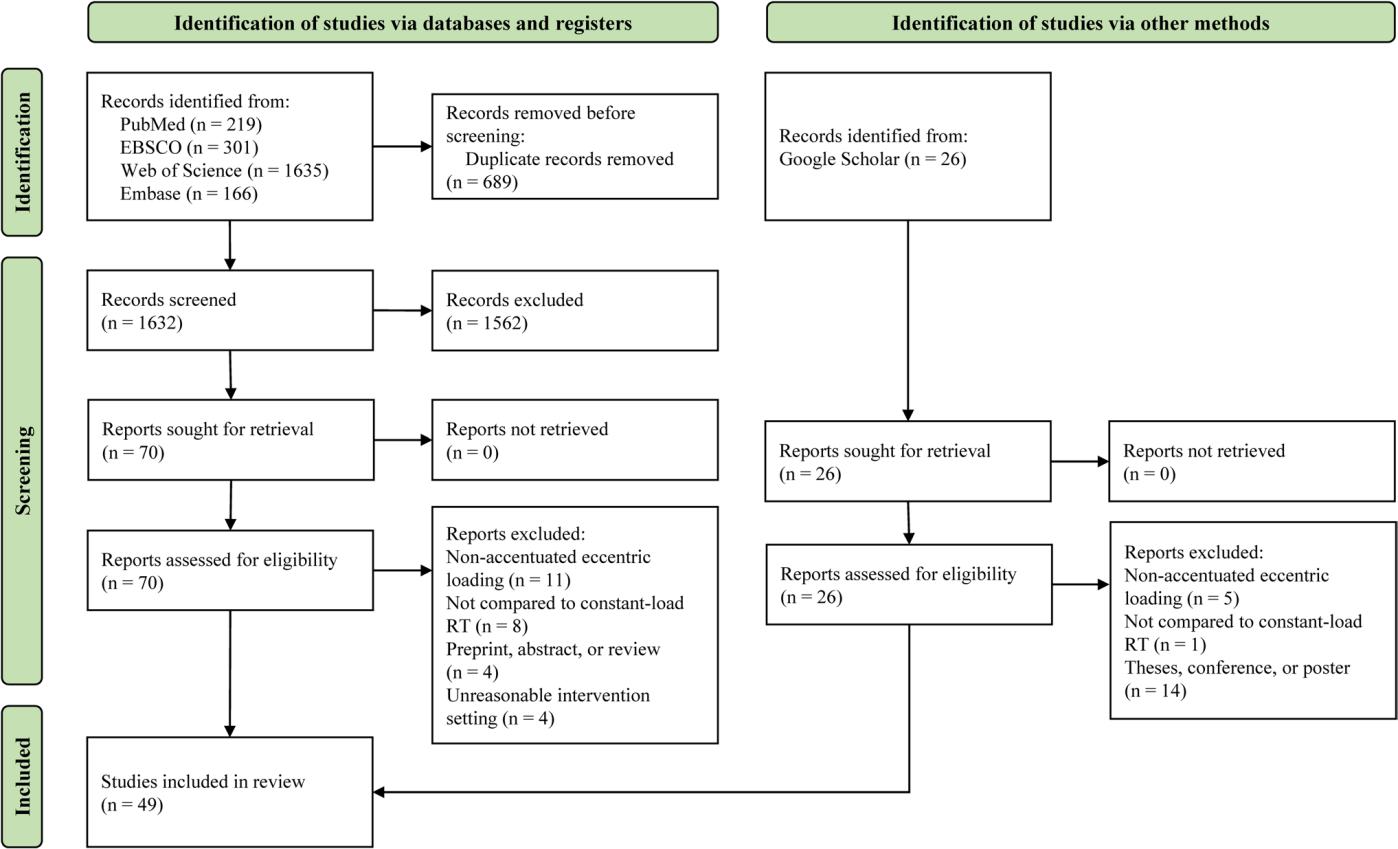
The study selection flow diagram of included and excluded research. *RT* resistance training

The flow diagram of the study selection process is shown in Fig. 1. The initial database search yielded a total of 2321 studies. After removing 689 duplicates, 1562 studies were excluded based on title and abstract screening. In the next phase, 26 studies were initially identified. Therefore, 96 studies were assessed for eligibility. Of these, 47 were excluded for reasons such as not using AEL, lacking a comparison to constant-load RT, inappropriate interventions, or being a thesis, preprint, review, abstract, poster, or conference document (see the electronic supplementary material, Supplementary 2). Ultimately, 49 studies were included in this systematic review and meta-analysis.

### Study Characteristics

Forty-nine studies that met the eligibility criteria were included in this systematic review and meta-analysis. Of these, 33 studies exclusively compared the acute effects of AEL, while 16 studies examined their chronic effects. The included participants totaled 773 young or adult healthy individuals, with no representation of elderly or other vulnerable populations. Additionally, most studies (*n* = 41) indicated that participants had varying levels of RT experience, with only eight studies reporting that participants had no RT experience or had not engaged in RT within the last 0.5 years [58–65]. Regarding training design, most studies (*n* = 34) used the same training prescription for both AEL and constant-load RT, including the same sets, repetitions, and concentric loads. However, 15 studies applied different training prescriptions between the two methods, primarily to balance the volume load [20–22, 58, 61, 63, 65–73]. Regarding the methods used to achieve AEL, most studies (*n* = 23) utilized weight releasers, while others employed techniques such as electrical motor, manual, pneumatic assistance or electromagnetic resistance. One study did not provide sufficient information to determine the method used for achieving AEL [29]. The outcomes included in this review were maximal dynamic strength (concentric and eccentric 1RM), concentric mechanical performance at submaximal loads (mean velocity, peak velocity, mean power, and peak power), unloaded countermovement jump height, maximal voluntary isometric force (peak force and/or peak torque), heart rate, acute muscle swelling, growth hormone, cortisol, creatine kinase, testosterone, lactate, electromyography, rating of perceived exertion, muscle soreness, muscle cross-sectional area, fascicle length, and fascicle angle. A more detailed description of the included studies can be found in Supplementary 3 (see the electronic supplementary material).

### Risk of Bias Assessment

**Fig. 2:**
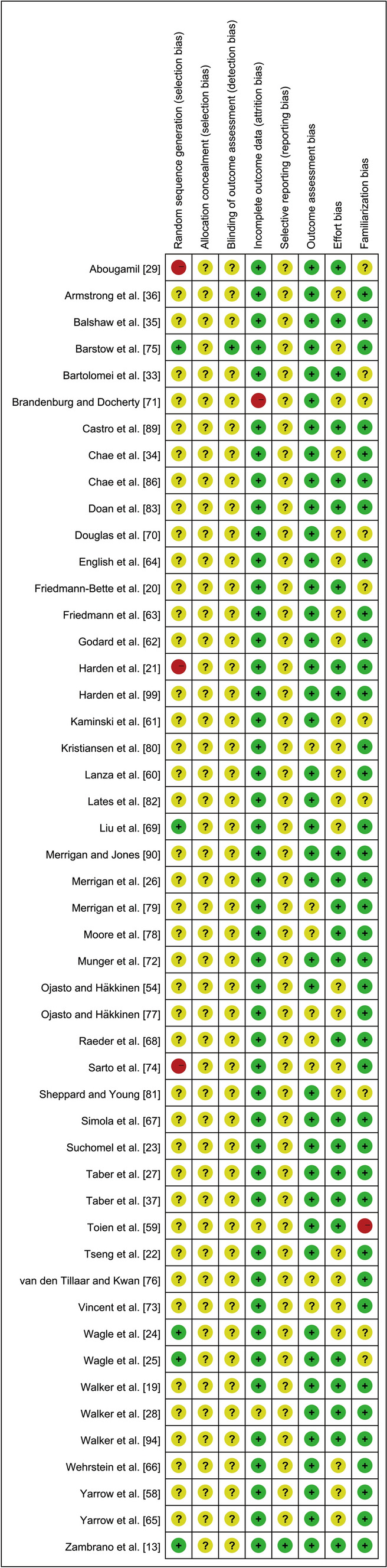
Risk of bias assessment of included studies (green circles represent low risk, yellow circles represent unclear risk, and red circles represent high risk)

Based on the assessment of selection bias, three studies were categorized as having a high risk of order effects due to a lack of randomization (Fig. 2) [21, 29, 74]. Five studies were categorized as low risk [13, 24, 25, 69, 75], while the remaining studies were classified as unclear risk. Regarding allocation concealment for participants and investigators, all studies lacked sufficient detailed information to allow for a proper judgment, so they were classified as unclear risk. In terms of detection bias, one study was categorized as low risk [75], while the remaining studies were classified as unclear risk. In terms of attrition bias, one study was rated high risk due to intervention-related, unbalanced dropout between groups that could bias the conclusions [71], two studies were rated unclear [28, 59], and the remaining studies were categorized as low risk. In terms of reporting bias, one study was rated low risk [13], whereas the remaining studies were classified as unclear risk because they either were not preregistered or their protocols lacked sufficient information to assess reporting bias. Regarding outcome assessment bias, eight studies were categorized as unclear risk because at least one measurement device had undetermined validity [68, 73, 74, 76–80]. The remaining studies were categorized as low risk. In terms of effort bias, 25 studies were classified as low risk, and the remaining 24 were rated unclear. In terms of familiarization bias, one study was rated high risk because the authors did not report a familiarization session and the participants had no RT experience [59]. Ten studies were rated unclear [20, 24, 25, 29, 33, 61, 70, 71, 81, 82], and the remaining studies were rated low risk.

### Meta-Analysis

#### Acute Responses

##### Mechanical Variables

*Maximal Dynamic Strength (Concentric 1RM [During])* There was no significant difference in the concentric maximum loads lifted between the AEL and constant-load conditions (SMD =  − 0.16 [95% CI − 0.59 to 0.28]; *p* = 0.48) (Table 1), with all studies using supramaximal AEL.

**Table 1:** Summary of meta-analysis and quality of evidence synthesis of mechanical variables

Outcome	Meta-analysis						GRADE				
	*k*	SMD	95% CI	95% PI	*p*	*I*^2^	1	2	3	4	Quality
Concentric 1RM (during)											
Accentuated eccentric loading	4	− 0.16	− 0.59 to 0.28	− 1.11 to 0.80	0.48	0%	None	− 1	None	None	Moderate
Supramaximal subgroup	4	− 0.16	− 0.59 to 0.28	− 1.11 to 0.80	0.48	0%	None	− 1	None	None	Moderate
CMPSL (during)											
Accentuated eccentric loading	29	− 0.07	− 0.21 to 0.08	− 0.21 to 0.08	0.37	0%	None	None	None	None	High
Submaximal subgroup	8	− 0.03	− 0.31 to 0.25	− 0.38 to 0.32	0.84	0%	None	None	None	None	High
Maximal subgroup	6	− 0.13	− 0.42 to 0.17	− 0.55 to 0.29	0.41	0%	None	None	None	None	High
Supramaximal subgroup	15	− 0.06	− 0.26 to 0.14	− 0.33 to 0.21	0.57	4%	None	None	None	None	High
Countermovement jump height (immediate)											
Accentuated eccentric loading	6	− 0.06	− 0.69 to 0.58	− 2.00 to 1.89	0.86	64%	None	None	None	None	High
Submaximal subgroup	2	0.25	− 3.04 to 3.54	*k* < 3	0.88	92%	None	− 1	− 1	None	Low
Maximal subgroup	1	–	–	–	–	–	–	–	–	–	–
Supramaximal subgroup	3	− 0.19	− 0.69 to 0.30	− 3.38 to 2.99	0.44	0%	None	− 1	None	None	Moderate
Countermovement jump height (delayed)											
Accentuated eccentric loading	2	− 0.23	− 0.81 to 0.35	*k* < 3	0.44	0%	None	− 1	None	None	Moderate
Maximal subgroup	1	–	–	–	–	–	–	–	–	–	–
Supramaximal subgroup	1	–	–	–	–	–	–	–	–	–	–
MVIF (immediate)											
Accentuated eccentric loading	4	0.03	− 0.37 to 0.43	− 0.85 to 0.90	0.89	0%	None	− 1	None	None	Moderate
Maximal subgroup	2	− 0.11	− 0.62 to 0.41	*k* < 3	0.68	0%	None	− 1	None	None	Moderate
Supramaximal subgroup	2	0.23	− 0.41 to 0.87	*k* < 3	0.48	3%	None	− 1	None	None	Moderate

A positive SMD indicates higher values for accentuated eccentric loading, while a negative SMD indicates higher values for constant-load resistance training

*1* risk of bias, *2* imprecision, *3* inconsistency, *4* indirectness, *CI* confidence interval, *CMPSL* concentric mechanical performance at submaximal loads, *GRADE* Grading of Recommendations Assessment, Development, and Evaluation, *k* number of trials, *MVIF* maximal voluntary isometric force, *PI* prediction interval, *RM* repetition maximum, *SMD* standardized mean difference

*Concentric Mechanical Performance at Submaximal Loads (During)* No significant differences in concentric mechanical performance at submaximal loads were observed between AEL and constant-load RT (SMD =  − 0.07 [95% CI − 0.21 to 0.08]; *p* = 0.37) (Table 1). Similar findings were noted in the submaximal (SMD =  − 0.03 [95% CI − 0.31 to 0.25]; *p* = 0.84), maximal (SMD =  − 0.13 [95% CI − 0.42 to 0.17]; *p* = 0.41), and supramaximal (SMD =  − 0.06 [95% CI − 0.26 to 0.14]; *p* = 0.57) subgroups.

*Countermovement Jump Height (Immediate and Delayed)* The immediate and delayed effects on countermovement jump height did not significantly differ between AEL and constant-load RT (immediate: SMD =  − 0.06 [95% CI − 0.69 to 0.58]; *p* = 0.86; delayed: SMD =  − 0.23 [95% CI − 0.81 to 0.35]; *p* = 0.44) (Table 1). Similarly, no significant difference was found in immediate effects between constant-load RT and either submaximal (SMD = 0.25 [95% CI − 3.04 to 3.54]; *p* = 0.88) or supramaximal (SMD =  − 0.19 [95% CI − 0.69 to 0.30]; *p* = 0.44) AEL protocols.

*Maximal Voluntary Isometric Force (Immediate)* The pooled analysis did not reveal a significant difference in the immediate effect on maximal voluntary isometric force between AEL and constant-load RT (SMD = 0.03 [95% CI − 0.37 to 0.43]; *p* = 0.89) (Table 1). Similar results were found in both the maximal (SMD =  − 0.11 [95% CI − 0.62 to 0.41]; *p* = 0.68) and supramaximal (SMD = 0.23 [95% CI − 0.41 to 0.87]; *p* = 0.48) subgroups.

##### Physiological Variables

*Lactate (During and Immediate)* No significant difference in blood lactate concentration during the intervention was observed between AEL and constant-load RT (SMD =  − 0.06 [95% CI − 0.48 to 0.36]; *p* = 0.78) (Table 2). A similar finding was noted in the supramaximal subgroup (SMD =  − 0.29 [95% CI − 0.86 to 0.28]; *p* = 0.32). In terms of immediate response post-intervention, AEL resulted in significantly higher blood lactate concentration compared to constant-load RT (SMD = 0.44 [95% CI 0.04–0.85]; *p* = 0.03) (Table 2). Similar findings were observed in the submaximal (SMD = 0.76 [95% CI 0.14–1.38]; *p* = 0.02), but not in the maximal subgroup (SMD = 0.74 [95% CI 0.00–1.49]; *p* = 0.05) or the supramaximal subgroup (SMD = 0.00 [95% CI − 0.51 to 0.52]; *p* = 0.99).

**Table 2:** Summary of meta-analysis and quality of evidence synthesis of physiological variables

Outcome	Meta-analysis						GRADE				
	*k*	SMD	95% CI	95% PI	*p*	*I*^2^	1	2	3	4	Quality
Lactate (during)											
Accentuated eccentric loading	3	− 0.06	− 0.48 to 0.36	− 2.79 to 2.66	0.78	0%	None	− 1	None	None	Moderate
Maximal subgroup	1	–	–	–	–	–	–	–	–	–	–
Supramaximal subgroup	2	− 0.29	− 0.86 to 0.28	*k* < 3	0.32	0%	None	− 1	None	None	Moderate
Lactate (immediate)											
Accentuated eccentric loading	10	0.44	0.04 to 0.85	− 0.78 to 1.66	0.03	56%	None	None	None	None	High
Submaximal subgroup	2	0.76	0.14 to 1.38	*k* < 3	0.02	0%	None	− 1	None	None	Moderate
Maximal subgroup	4	0.74	0.00 to 1.49	− 2.38 to 3.87	0.05	68%	None	None	None	None	High
Supramaximal subgroup	4	0.00	− 0.51 to 0.52	− 1.70 to 1.71	0.99	31%	None	− 1	None	None	Moderate
Testosterone (immediate)											
Accentuated eccentric loading	3	0.68	− 0.25 to 1.61	− 9.77 to 11.13	0.15	67%	None	− 1	None	None	Moderate
Maximal subgroup	1	–	–	–	–	–	–	–	–	–	–
Supramaximal subgroup	2	0.82	− 0.81 to 2.46	*k* < 3	0.32	82%	None	− 1	− 1	None	Low
Growth hormone (immediate)											
Accentuated eccentric loading	6	0.50	0.15 to 0.86	− 0.00 to 1.01	0.01	0%	None	None	None	None	High
Submaximal subgroup	2	0.53	− 0.14 to 1.20	*k* < 3	0.12	18%	None	− 1	None	None	Moderate
Maximal subgroup	2	0.55	− 0.06 to 1.15	*k* < 3	0.08	0%	None	− 1	None	None	Moderate
Supramaximal subgroup	2	0.42	− 0.22 to 1.06	*k* < 3	0.20	0%	None	− 1	None	None	Moderate
Creatine kinase (immediate)											
Accentuated eccentric loading	2	0.09	− 0.38 to 0.55	*k* < 3	0.72	0%	None	− 1	None	None	Moderate
Maximal subgroup	1	–	–	–	–	–	–	–	–	–	–
Creatine kinase (delayed)											
Accentuated eccentric loading	3	0.14	− 0.25 to 0.53	− 2.40 to 2.69	0.48	0%	None	None	None	None	High
Maximal subgroup	2	0.16	− 0.31 to 0.63	*k* < 3	0.51	0%	None	− 1	None	None	Moderate
Cortisol (immediate)											
Accentuated eccentric loading	3	0.39	− 0.01 to 0.78	− 2.17 to 2.95	0.05	0%	None	None	None	None	High
Supramaximal subgroup	3	0.39	− 0.01 to 0.78	− 2.17 to 2.95	0.05	0%	None	None	None	None	High
Heart rate (during)											
Accentuated eccentric loading	3	1.18	− 0.11 to 2.47	− 14.67 to 17.04	0.07	86%	None	− 1	− 1	None	Low
Maximal subgroup	1	–	–	–	–	–	–	–	–	–	–
Supramaximal subgroup	2	0.52	− 0.05 to 1.10	*k* < 3	0.07	0%	None	− 1	None	None	Moderate
Acute muscle swelling (immediate)											
Accentuated eccentric loading	3	0.26	− 0.37 to 0.88	− 6.46 to 6.98	0.42	59%	None	None	None	None	High
Supramaximal subgroup	3	0.26	− 0.37 to 0.88	− 6.46 to 6.98	0.42	59%	None	None	None	None	High
Concentric electromyography (during)											
Accentuated eccentric loading	17	− 0.01	− 0.21 to 0.18	− 0.23 to 0.20	0.90	0%	None	None	None	None	High
Submaximal subgroup	6	0.01	− 0.31 to 0.33	− 0.45 to 0.46	0.96	0%	None	None	None	None	High
Maximal subgroup	2	− 0.14	− 0.71 to 0.43	*k* < 3	0.63	0%	None	− 1	None	None	Moderate
Supramaximal subgroup	9	0.00	− 0.28 to 0.28	− 0.34 to 0.34	0.99	0%	None	None	None	None	High
Eccentric electromyography (during)											
Accentuated eccentric loading	16	0.37	0.09 to 0.65	− 0.49 to 1.23	0.01	44%	None	None	None	None	High
Submaximal subgroup	6	0.24	− 0.24 to 0.72	− 1.16 to 1.63	0.33	53%	None	None	None	None	High
Maximal subgroup	2	0.90	− 0.15 to 1.95	*k* < 3	0.09	66%	None	− 1	None	None	Moderate
Supramaximal subgroup	8	0.33	− 0.02 to 0.69	− 0.47 to 1.14	0.07	28%	None	None	None	None	High

A positive SMD indicates higher values for accentuated eccentric loading, while a negative SMD indicates higher values for constant-load resistance training

*1* risk of bias, *2* imprecision, *3* inconsistency, *4* indirectness, *CI* confidence interval, *GRADE* Grading of Recommendations Assessment, Development, and Evaluation, *k* number of trials, *PI* prediction interval, *SMD* standardized mean difference

*Testosterone (Immediate)* There was no significant difference in the immediate effect on testosterone concentration between AEL and constant-load RT (SMD = 0.68 [95% CI − 0.25 to 1.61]; *p* = 0.15) (Table 2). A similar result was found in the supramaximal subgroup (SMD = 0.82 [95% CI − 0.81 to 2.46]; *p* = 0.32).

*Growth Hormone (Immediate)* The pooled analysis demonstrated that AEL resulted in a significantly higher immediate effect on growth hormone concentration compared to constant-load RT (SMD = 0.50 [95% CI 0.15–0.86]; *p* = 0.01) (Table 2). However, no significant differences were observed in the submaximal (SMD = 0.53 [95% CI − 0.14 to 1.20]; *p* = 0.12), maximal (SMD = 0.55 [95% CI − 0.06 to 1.15]; *p* = 0.08), or supramaximal (SMD = 0.42 [95% CI − 0.22 to 1.06]; *p* = 0.20) subgroups.

*Creatine Kinase (Immediate and Delayed)* Our pooled analysis showed no significant difference in creatine kinase concentration between AEL and constant-load RT, either for immediate (SMD = 0.09 [95% CI − 0.38 to 0.55]; *p* = 0.72) or delayed (SMD = 0.14 [95% CI − 0.25 to 0.53]; *p* = 0.48) effects (Table 2). A similar result was observed in the maximal AEL subgroup for the delayed effect (SMD = 0.16 [95% CI − 0.31 to 0.63]; *p* = 0.51).

*Cortisol (Immediate)* The immediate effect on cortisol concentration did not significantly differ between AEL and constant-load RT (SMD = 0.39 [95% CI − 0.01 to 0.78]; *p* = 0.05) (Table 2), with all studies using supramaximal AEL.

*Heart Rate (During)* No significant difference in heart rate during the intervention was observed between AEL and constant-load RT (SMD = 1.18 [95% CI − 0.11 to 2.47]; *p* = 0.07) (Table 2). A similar finding was also noted in the supramaximal subgroup (SMD = 0.52 [95% CI − 0.05 to 1.10]; *p* = 0.07).

*Acute Muscle Swelling (Immediate)* The pooled analysis did not reveal a significant difference in the immediate effect on muscle swelling between AEL and constant-load RT (SMD = 0.26 [95% CI − 0.37 to 0.88]; *p* = 0.42) (Table 2), with all studies using supramaximal AEL.

*Concentric Electromyography (During)* Our pooled analysis revealed no significant difference in muscle electrical activity during the concentric phase between AEL and constant-load RT (SMD =  − 0.01 [95% CI − 0.21 to 0.18]; *p* = 0.90) (Table 2). Similar findings were observed in the submaximal (SMD = 0.01 [95% CI − 0.31 to 0.33]; *p* = 0.96), maximal (SMD =  − 0.14 [95% CI − 0.71 to 0.43]; *p* = 0.63), and supramaximal (SMD = 0.00 [95% CI − 0.28 to 0.28]; *p* = 0.99) subgroups.

*Eccentric Electromyography (During)* The pooled analysis indicated that AEL resulted in significantly higher muscle electrical activity during the eccentric phase compared to constant-load RT (SMD = 0.37 [95% CI 0.09–0.65]; *p* = 0.01) (Table 2). However, no significant differences were observed in the submaximal (SMD = 0.24 [95% CI − 0.24 to 0.72]; *p* = 0.33), maximal (SMD = 0.90 [95% CI − 0.15 to 1.95]; *p* = 0.09), and supramaximal (SMD = 0.33 [95% CI − 0.02 to 0.69]; *p* = 0.07) subgroups.

##### Perceptual Variables

*Rating of Perceived Exertion (Immediate)* The pooled analysis indicated that AEL induced a significantly higher rating of perceived exertion compared to constant-load RT (SMD = 1.72 [95% CI 0.89–2.56]; *p* = 0.01) (Table 3). Similar findings were observed in both the maximal (SMD = 1.53 [95% CI 0.53–2.53]; *p* = 0.01) and supramaximal (SMD = 2.17 [95% CI 0.31–4.02]; *p* = 0.02) subgroups.

**Table 3:** Summary of meta-analysis and quality of evidence synthesis of perceptual variables

Outcome	Meta-analysis						GRADE				
	*k*	SMD	95% CI	95% PI	*p*	*I*^2^	1	2	3	4	Quality
Rating of perceived exertion (immediate)											
Accentuated eccentric loading	7	1.72	0.89 to 2.56	− 1.07 to 4.52	0.01	82%	None	None	− 1	None	Moderate
Maximal subgroup	4	1.53	0.53 to 2.53	− 3.00 to 6.06	0.01	82%	None	None	− 1	None	Moderate
Supramaximal subgroup	3	2.17	0.31 to 4.02	− 20.53 to 24.86	0.02	88%	None	− 1	− 1	None	Low
Muscle soreness (immediate)											
Accentuated eccentric loading	2	0.28	− 0.25 to 0.81	*k* < 3	0.30	0%	None	− 1	None	None	Moderate
Maximal subgroup	1	–	–	–	–	–	–	–	–	–	–
Supramaximal subgroup	1	–	–	–	–	–	–	–	–	–	–
Muscle soreness (delayed)											
Accentuated eccentric loading	4	0.18	− 0.15 to 0.52	− 0.55 to 0.91	0.28	0%	None	None	None	None	High
Maximal subgroup	1	–	–	–	–	–	–	–	–	–	–
Supramaximal subgroup	3	0.05	− 0.34 to 0.44	− 2.49 to 2.60	0.79	0%	None	None	None	None	High

A positive SMD indicates higher values for accentuated eccentric loading, while a negative SMD indicates higher values for constant-load resistance training

*1* risk of bias, *2* imprecision, *3* inconsistency, *4* indirectness, *CI* confidence interval, *GRADE* Grading of Recommendations Assessment, Development, and Evaluation, *k* number of trials, *PI* prediction interval, *SMD* standardized mean difference

*Muscle Soreness (Immediate and Delayed)* The immediate and delayed effects on muscle soreness did not significantly differ between AEL and constant-load RT (immediate: SMD = 0.28 [95% CI − 0.25 to 0.81]; *p* = 0.30, delayed: SMD = 0.18 [95% CI − 0.15 to 0.52]; *p* = 0.28) (Table 3). Similarly, no significant difference in delayed muscle soreness was observed between supramaximal AEL and constant-load RT (SMD = 0.05 [95% CI − 0.34 to 0.44]; *p* = 0.79).

#### Chronic Adaptations

##### Strength-Related Adaptations

*Maximal Dynamic Strength (Concentric and Eccentric 1RM)* The pooled analysis did not reveal a significant difference in concentric 1RM between AEL and constant-load RT (SMD = 0.12 [95% CI − 0.17 to 0.41]; *p* = 0.41) (Table 4). A similar result was also observed in the supramaximal subgroup (SMD = 0.04 [95% CI − 0.28 to 0.36]; *p* = 0.81). There was no significant difference in eccentric 1RM between AEL and constant-load RT (SMD = 0.19 [95% CI − 0.48 to 0.85]; *p* = 0.58).

**Table 4:** Summary of meta-analysis and quality of evidence synthesis of strength-related adaptations

Outcome	Meta-analysis						GRADE				
	*k*	SMD	95% CI	95% PI	*p*	*I*^2^	1	2	3	4	Quality
Concentric 1RM											
Accentuated eccentric loading	10	0.12	− 0.17 to 0.41	− 0.22 to 0.46	0.41	0%	None	None	None	None	High
Maximal subgroup	1	–	–	–	–	–	–	–	–	–	–
Supramaximal subgroup	8	0.04	− 0.28 to 0.36	− 0.36 to 0.44	0.81	0%	None	None	None	None	High
Eccentric 1RM											
Accentuated eccentric loading	2	0.19	− 0.48 to 0.85	*k* < 3	0.58	0%	None	− 1	None	None	Moderate
Supramaximal subgroup	1	–	–	–	–	–	–	–	–	–	–
MVIF											
Accentuated eccentric loading	2	0.03	− 0.62 to 0.67	*k* < 3	0.93	0%	None	− 1	None	None	Moderate
Supramaximal subgroup	2	0.03	− 0.62 to 0.67	*k* < 3	0.93	0%	None	− 1	None	None	Moderate
Countermovement jump height											
Accentuated eccentric loading	2	0.04	− 0.48 to 0.57	*k* < 3	0.87	0%	− 1	− 1	None	None	Low
Supramaximal subgroup	2	0.04	− 0.48 to 0.57	*k* < 3	0.87	0%	− 1	− 1	None	None	Low

A positive SMD indicates higher values for accentuated eccentric loading, while a negative SMD indicates higher values for constant-load resistance training

*1* risk of bias, *2* imprecision, *3* inconsistency, *4* indirectness, *CI* confidence interval, *GRADE* Grading of Recommendations Assessment, Development, and Evaluation, *k* number of trials, *MVIF* maximal voluntary isometric force, *PI* prediction interval, *RM* repetition maximal, *SMD* standardized mean difference

*Maximal Voluntary Isometric Force* Our pooled analysis indicated no significant difference in maximal voluntary isometric force between AEL and constant-load RT (SMD = 0.03 [95% CI − 0.62 to 0.67]; *p* = 0.93) (Table 4), with all studies using supramaximal AEL.

*Countermovement Jump Height* The pooled analysis demonstrated no significant difference in countermovement jump height between AEL and constant-load RT (SMD = 0.04 [95% CI − 0.48 to 0.57]; *p* = 0.87) (Table 4), with all studies using supramaximal AEL.

##### Structural Adaptations

*Fascicle Angle* Our pooled analysis did not reveal a significant difference in fascicle angle between AEL and constant-load RT (SMD =  − 0.10 [95% CI − 0.77 to 0.57]; *p* = 0.77) (Table 5), with all studies using supramaximal AEL.

**Table 5:** Summary of meta-analysis and quality of evidence synthesis of structural adaptations

Outcome	Meta-analysis						GRADE				
	*k*	SMD	95% CI	95% PI	*p*	*I*^2^	1	2	3	4	Quality
Fascicle angle											
Accentuated eccentric loading	2	− 0.10	− 0.77 to 0.57	*k* < 3	0.77	0%	None	− 1	None	None	Moderate
Supramaximal subgroup	2	− 0.10	− 0.77 to 0.57	*k* < 3	0.77	0%	None	− 1	None	None	Moderate
Fascicle length											
Accentuated eccentric loading	2	0.90	− 0.39 to 2.19	*k* < 3	0.17	68%	None	− 1	None	None	Moderate
Supramaximal subgroup	2	0.90	− 0.39 to 2.19	*k* < 3	0.17	68%	None	− 1	None	None	Moderate
Muscle cross-sectional area											
Accentuated eccentric loading	3	− 0.06	− 0.59 to 0.48	− 3.54 to 3.42	0.84	0%	None	− 1	None	None	Moderate
Submaximal subgroup	1	–	–	–	–	–	–	–	–	–	–
Supramaximal subgroup	2	− 0.12	− 0.76 to 0.51	*k* < 3	0.70	0%	− 1	− 1	None	None	Low

A positive SMD indicates higher values for accentuated eccentric loading, while a negative SMD indicates higher values for constant-load resistance training

*1* risk of bias, *2* imprecision, *3* inconsistency, *4* indirectness, *CI* confidence interval, *GRADE* Grading of Recommendations Assessment, Development, and Evaluation, *k* number of trials, *PI* prediction interval, *SMD* standardized mean difference

*Fascicle Length* The pooled analysis indicated no significant difference in fascicle length between AEL and constant-load RT (SMD = 0.90 [95% CI − 0.39 to 2.19]; *p* = 0.17) (Table 5), with all studies using supramaximal AEL.

*Muscle Cross-Sectional Area* No significant difference in muscle cross-sectional area was observed between AEL and constant-load RT (SMD =  − 0.06 [95% CI − 0.59 to 0.48]; *p* = 0.84) (Table 5). A similar finding was noted in the supramaximal subgroup (SMD =  − 0.12 [95% CI − 0.76 to 0.51]; *p* = 0.70).

### Publication Bias

Publication bias was assessed for outcomes with ten or more available studies (acute variables: concentric mechanical performance at submaximal loads, blood lactate, electromyography during the concentric phase, and electromyography during the eccentric phase; chronic variable: concentric 1RM). The results of the publication bias analysis are presented in Supplementary 4 (see the electronic supplementary material). Notably, publication bias was observed in the concentric mechanical performance at submaximal loads (*p* = 0.02) and electromyography during the eccentric phase (*p* = 0.02).

### Sensitivity Analysis

The sensitivity analysis is presented in Supplementary 5 (see the electronic supplementary material). According to the results, there was no significant difference in the immediate effect on blood lactate concentration between AEL and constant-load RT (sensitivity analysis: SMD = 0.30 [95% CI − 0.22 to 0.81]; *p* = 0.26), which differs from the pooled analysis (pooled analysis: SMD = 0.44 [95% CI 0.04–0.85]; *p* = 0.03). However, the significance of the remaining outcomes did not change. Notably, for some outcomes (e.g., acute concentric 1RM), the sensitivity analysis yielded the same number of trials, SMD, CI, and PI as those reported in Tables 1, 2, 3, 4, and 5 because all included studies used matched training prescriptions between AEL and constant-load RT, leaving no studies to exclude. In contrast, some outcomes (e.g., acute countermovement jump height) could not be analyzed in the sensitivity analysis because excluding non-equivalent training prescriptions left too few studies.

## Discussion

**Fig. 3:**
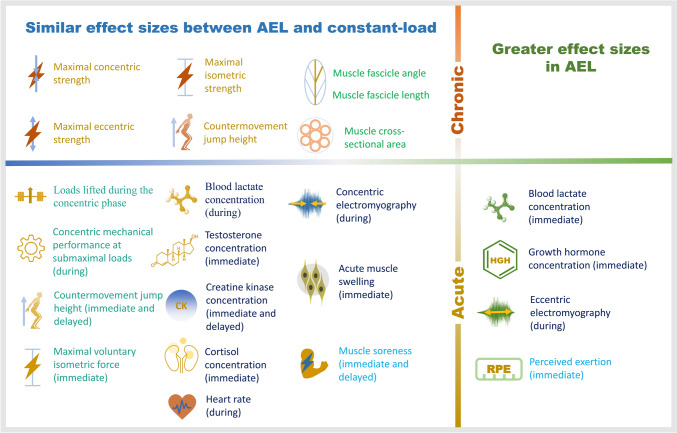
Comparison of acute responses and chronic adaptations between accentuated eccentric loading (AEL) and constant-load resistance training. *CK* creatine kinase, *RPE* rating of perceived exertion

This is the first systematic review and meta-analysis to compare AEL and constant-load RT in terms of acute effects on mechanical, metabolic, and perceptual variables, as well as chronic effects on strength and structural adaptations. Our findings demonstrated the following, in terms of acute responses: (1) AEL resulted in similar loads lifted during the concentric phases, concentric mechanical performance at submaximal loads (during), countermovement jump height (immediate and delayed), and maximal voluntary isometric force (immediate) compared to constant-load RT; (2) AEL led to higher blood lactate concentration (immediate), growth hormone concentration (immediate), and eccentric electromyography (during) than constant-load RT, but similar blood lactate concentration (during), testosterone concentration (immediate), creatine kinase concentration (immediate and delayed), cortisol concentration (immediate), heart rate (during), acute muscle swelling (immediate), and concentric electromyography (during); (3) AEL resulted in a higher rating of perceived exertion (immediate) compared to constant-load RT, while showing similar levels of muscle soreness (immediate and delayed). In terms of chronic adaptations, (4) AEL induced similar adaptations to constant-load RT in maximal concentric strength, maximal eccentric strength, maximal isometric strength, and countermovement jump height; (5) AEL also led to similar adaptations as constant-load RT in muscle fascicle angle, muscle fascicle length, and muscle cross-sectional area (Fig. 3). In conclusion, AEL can be recommended for individuals aiming to augment within-training eccentric muscle activation. However, it is important to note that implementing AEL may elevate metabolic responses and perceived exertion during training. Therefore, to mitigate the acute and short-term changes in fatigue, longer and more frequent inter-set rest periods and extended recovery between sessions should be considered. Additionally, it should be acknowledged that there is currently a lack of compelling evidence that AEL leads to enhanced chronic training adaptations. Thus, practitioners need to carefully consider and justify the implementation of this training method within a training cycle.

### Acute Responses

#### Maximal Dynamic Strength

Our finding suggested that AEL did not allow participants to lift greater maximal loads during the concentric phase when compared to constant-load RT (SMD =  − 0.16;* p* = 0.48). In this review, two studies examined the impact of AEL on 1RM tests [77, 83]. One study evaluated eccentric loads of 105%, 110%, and 120% 1RM in the bench press, finding that higher eccentric loads impaired concentric performance, with the 120% load being the most detrimental [77]. However, this study tested constant-load 1RM before performing an AEL 1RM test in the same session, likely introducing fatigue that compromised the second test's results. In contrast, Doan et al. [83] employed a more robust design, with randomized and separate sessions for constant-load and AEL 1RM tests. Their findings demonstrated that a load of 105% 1RM during the eccentric phase enhanced the bench press concentric 1RM. Thus, incorporating an additional 5% eccentric load during the 1RM test may help enhance athletes’ maximal dynamic concentric strength in the bench press.

#### Concentric Mechanical Performance at Submaximal Loads

In this meta-analysis, we used proxy variables—mean velocity, peak velocity, mean power, and peak power—to assess the effect of AEL on concentric mechanical performance at submaximal loads. Our results indicated that AEL led to similar concentric mechanical performance at submaximal loads when compared to constant-load RT (SMD =  − 0.07; *p* = 0.37). Theoretically, additional load during the eccentric phase should increase the elastic energy stored in tendons, thereby enhancing subsequent concentric performance [84]. For instance, Aboodarda et al. [85] observed improved concentric performance in the countermovement jump with an additional load of 30% body weight during the eccentric phase. However, in RT, larger eccentric loads (50–120% 1RM) are typically applied, which may force individuals to adopt slower pacing strategies during the eccentric and amortization phases, thereby reducing the potentiation effects of the additional eccentric load [30]. Another plausible explanation is that the additional load in AEL results in greater muscle fatigue, which may interfere with concentric performance [14]. Based on current evidence, AEL is not recommended for athletes aiming to improve concentric mechanical performance at submaximal loads.

It is worth noting that Chae et al. [86] reported positive effects of AEL on concentric peak velocity and peak power versus traditional constant-load RT. In their back-squat protocol, AEL was paired with a rest-redistribution strategy using a moderate concentric load (60% 1RM) and an eccentric overload (110% 1RM) via weight releasers. Each ten-rep set was partitioned into five within-set clusters of two reps (AEL + RR-5). Between clusters, participants re-racked the bar and took 15 s of intra-set rest, with 90 s of inter-set rest; the weight releasers were reattached during these rest periods. This design may have helped reduce fatigue accumulation and improve recovery within sets [49], which could explain the better concentric mechanical performance at submaximal loads observed in AEL. Additionally, Merrigan et al. [26] found that squat using AEL with an eccentric load of 120% 1RM only during the first repetition enhanced subsequent concentric performance within the set (concentric 65% 1RM). However, this effect diminished with higher concentric loads (concentric 80% 1RM with eccentric 120% 1RM). These two studies imply that when using AEL, employing rest redistribution strategies and carefully selecting the load used may enhance concentric mechanical performance at submaximal loads. However, as few studies have directly compared different AEL designs, our understanding of this topic remains limited. We recommend that further investigations are completed to elucidate the potential effects of rest redistribution methods with AEL.

#### Countermovement Jump Height and Maximal Voluntary Isometric Force

In principle, AEL would be expected to produce a greater fatigue response following intervention due to the additional eccentric loads. However, despite some uncertainty, as noted by the wide PIs (countermovement jump height [95% PI − 2.00 to 1.89]), our point estimates did not suggest that AEL reduced countermovement jump height (SMD =  − 0.06; *p* = 0.86) or maximal voluntary isometric force performance (SMD = 0.03; *p* = 0.89) immediately after the intervention compared to constant-load RT. Furthermore, Tseng et al. [22] and Raeder et al. [68] tracked the delayed effects of AEL on countermovement jump height and similarly found no difference between AEL and constant-load RT (SMD =  − 0.23; *p* = 0.44). The comparable immediate and delayed fatigue between the two approaches may be related to their training designs. In this review, six studies examined residual fatigue outcomes, with four employing lower concentric loads or fewer repetitions in the AEL condition [22, 67–69]. For example, Tseng et al. [22] used three sets of five repetitions with a concentric load of 85% 1RM in constant-load RT but three sets of four repetitions with a concentric load of 80% 1RM in the AEL condition. Additionally, one study, despite using the same repetitions and concentric loads for both AEL and constant-load RT, applied a rest redistribution strategy only in AEL [86], which also helped alleviate fatigue [49]. Therefore, when implementing AEL, coaches can help athletes manage residual fatigue from the additional eccentric load by adjusting repetitions, loads, and rest strategies.

#### Electromyography

Our findings indicated that AEL did not induce higher muscle electrical activity during the concentric phase compared to constant-load RT (SMD =  − 0.01; *p* = 0.90). However, AEL increased eccentric muscle electrical activity (SMD = 0.37; *p* = 0.01). This result is unsurprising, as the primary distinction between AEL and constant-load RT lies in the eccentric load, while the concentric phase typically involves similar loads in both methods. According to Henneman’s size principle of recruitment, muscle fibers are progressively recruited based on the force demands of the task [87]. As a result, the increased eccentric load in AEL requires the recruitment of more motor units, particularly higher-threshold motor units, to generate greater eccentric force [87, 88]. The elevated eccentric muscle electrical activity reflects this enhanced recruitment process. Therefore, AEL can be effectively applied to help improve athletes' eccentric muscle recruitment capacity.

In the subgroup analysis that analyzed changes in eccentric muscle electrical activity, all three types of AEL showed effect sizes favoring AEL (submaximal AEL: SMD = 0.24, *p* = 0.33; maximal AEL: SMD = 0.90, *p* = 0.09; supramaximal AEL: SMD = 0.33, *p* = 0.07), which is consistent with the pooled analysis. However, none of these results reached statistical significance. To investigate the lack of significance, we examined the studies that assessed eccentric muscle electrical activity within each subgroup. The submaximal subgroup had the smallest effect size (SMD = 0.24) among all subgroups, primarily due to the study by Ojasto and Häkkinen [77], which reported several small effect sizes. In this study, the authors compared four AEL conditions with constant-load RT (concentric 50% 1RM paired with eccentric 60%, 70%, 80%, and 90% 1RM vs. concentric 50% 1RM paired with eccentric 50% 1RM). The findings indicated that higher eccentric loads led to greater eccentric muscle electrical activity during the bench press. However, significant differences were observed only when the eccentric loads reached 80% 1RM or higher. This suggests that in AEL, the disparity between eccentric and concentric loads must surpass a certain threshold to induce significantly higher eccentric muscle electrical activity.

Although not statistically significant, the maximal subgroup exhibited a large difference in eccentric muscle electrical activity (SMD > 0.80). In this subgroup, two effect sizes were reported by Castro et al. [89]. The authors compared two AEL designs with two constant-load RT designs (concentric 30% 1RM with eccentric 30% 1RM vs. concentric 30% 1RM with eccentric 100% 1RM, and concentric 80% 1RM with eccentric 80% 1RM vs. concentric 80% 1RM with eccentric 100% 1RM). Their findings indicated that both AEL conditions resulted in increased eccentric muscle electrical activity. Given this, we speculate that the non-significant result may be attributed to the small sample size.

The effect size of the supramaximal subgroup (SMD = 0.33) was very similar to that for the pooled analysis. In this subgroup, the non-significant result was primarily influenced by the study of Ojasto and Häkkinen [77]. This study compared constant-load RT (concentric 100% 1RM with eccentric 100% 1RM) with three different AEL conditions (concentric 100% 1RM with eccentric 105%, 110%, and 120% 1RM) and found no significant differences between them. As previously mentioned, this outcome may be due to the study's methodological design, where constant-load RT was always performed before AEL within the same session. This likely induced fatigue during the constant-load RT, potentially compromising the results of the subsequent AEL performance.

#### Heart Rate

Our pooled analysis showed a similar heart rate response during interventions between AEL and constant-load RT (SMD = 1.18;* p* = 0.07), though there was considerable variability as noted by the wide PIs (95% PI − 14.67 to 17.04). In our review, two studies specifically examined the impact of AEL on heart rate, and both reported that AEL led to a higher heart rate compared to constant-load RT [34, 73]. Furthermore, Chae et al. [34] found that even when a rest redistribution strategy was employed, AEL still resulted in a significantly higher heart rate during the intervention compared to constant-load RT with a traditional set structure. This suggests that the additional eccentric load in AEL may impose greater cardiovascular demands. Therefore, AEL may not be suitable for individuals with cardiovascular health concerns, such as those with pre-existing heart conditions and patients with uncontrolled hypertension.

#### Lactate

Although there was some uncertainty in blood lactate concentration during interventions (95% PI − 2.79 to 2.66), the point estimate suggested that AEL did not induce a higher blood lactate response compared to constant-load RT (SMD =  − 0.06; *p* = 0.78). Regarding the immediate post-intervention response, the pooled analysis indicated a significantly higher blood lactate concentration in AEL (SMD = 0.44; *p* = 0.03). However, further sensitivity analysis—controlling for variables such as loads, sets, and repetitions per set—discovered a change in significance (SMD = 0.30; *p* = 0.26). Since sensitivity analysis reduces potential confounding factors, we believe its results offer a more accurate representation of AEL’s true effects. Thus, the additional eccentric load in AEL does not seem to result in higher blood lactate concentration. One possible explanation lies in the average load used in AEL. While AEL increases the eccentric load, the concentric load remains consistent with that of constant-load RT, potentially making the additional training stimulus less impactful than anticipated. However, it is worth noting that Ojasto and Häkkinen [54] compared constant-load RT (concentric 70% 1RM with eccentric 70% 1RM) with three AEL designs (concentric 70% 1RM with eccentric 80%, 90%, and 100% 1RM) and observed a trend: as the eccentric load increased, post-intervention blood lactate concentration also increased. Although the differences were not statistically significant even at an eccentric load of 100% 1RM, it can be speculated that sufficiently high additional eccentric loads in AEL might lead to significantly higher blood lactate concentration. Therefore, if athletes aim to avoid an excessive blood lactate response when using AEL, excessively high additional eccentric loads should be approached with caution (e.g., exceeding 30% 1RM of the additional eccentric load).

Interestingly, Chae et al. [34] reported that when AEL employed a rest redistribution strategy, taking 15-s rests after every two repetitions resulted in significantly lower blood lactate concentration compared to constant-load RT, even though the total rest time remained the same in both conditions. Thus, this rest redistribution strategy should be considered in AEL when the goal is to avoid excessive blood lactate responses, particularly when applying high eccentric loads.

#### Muscle Damage

The pooled analysis suggested that AEL and constant-load RT resulted in similar levels of muscle damage immediately post-intervention (creatine kinase: SMD = 0.09; *p* = 0.72; acute muscle swelling: SMD = 0.26;* p* = 0.42), although considerable variability was observed in acute muscle swelling (95% PI − 6.46 to 6.98). For delayed muscle damage, despite the substantial variability (creatine kinase: 95% PI − 2.40 to 2.69), the point estimate indicated no significant differences between AEL and constant-load RT (creatine kinase: SMD = 0.14; *p* = 0.48). In this review, five studies examined the impact of AEL on muscle damage [33, 66, 68, 73, 90], with only Bartolomei et al. [33] reporting that AEL caused more severe muscle damage than constant-load RT, as evidenced by more pronounced acute muscle swelling post-intervention. To investigate the reasons for this discrepancy, we analyzed the study designs of the five included studies [33, 66, 68, 73, 90]. It was observed that Bartolomei et al. [33] utilized a higher load (AEL: concentric 80% 1RM with eccentric 120% 1RM; constant-load: concentric 80% 1RM) combined with greater training volume (30 repetitions). This combination likely resulted in AEL accumulating a more substantial high-intensity training volume, which contributed to the observed increase in muscle damage [91, 92]. Therefore, when implementing AEL, avoiding the combination of high intensity and high-volume protocols may mitigate excessive muscle damage.

#### Hormone Response

Our findings indicated no significant difference in cortisol response between AEL and constant-load RT post-intervention (SMD = 0.39; *p* = 0.05), with substantial variability in the results (95% PI − 2.17 to 2.95). Notably, one study by Merrigan and Jones [90] reported a reduced cortisol concentration following AEL compared with pre-test levels, which the authors attributed to a smaller training stimulus [93]. In this study, participants performed three sets of five repetitions with concentric 65% 1RM and eccentric 120% 1RM, as well as three sets of three repetitions with concentric 80% 1RM and eccentric 120% 1RM, and AEL was applied only to the first repetition of each set. In contrast, another study that observed a significantly higher post-intervention cortisol response with AEL used three sets performed to failure with concentric loads of approximately 75–85% 1RM and eccentric loads 40% greater than the concentric loads [94]. Therefore, when implementing AEL, coaches can regulate athletes’ physiological stress responses by managing the total training load.

The pooled analysis demonstrated that AEL generated a similar testosterone response post-intervention compared to constant-load RT (SMD = 0.68; *p* = 0.15), with wide PIs (95% PI − 9.77 to 11.13). However, regarding growth hormone response, AEL elicits a significantly higher post-intervention response compared to constant-load RT (SMD = 0.50; *p* = 0.01). This result remained consistent even after controlling for variables such as loads, sets, and repetitions per set (sensitivity analysis: SMD = 0.50; *p* = 0.03). Theoretically, testosterone and growth hormone are load responsive; given equivalent training volume, the higher eccentric load characteristic of AEL would be expected to augment both [95–97]. However, our findings only discovered a higher growth hormone response in the AEL condition. This discrepancy may be attributed to the training designs of the included studies. In this review, three studies compared testosterone responses between AEL and constant-load RT [58, 65, 94]. Among these, two studies employed a lower training volume in the AEL condition compared to constant-load RT [58, 65]. For instance, in the study by Yarrow et al. [65], the constant-load RT condition included four sets of six repetitions, while the AEL condition only included three sets of six repetitions. This difference in training volume likely reduced the overall training stimulus in AEL, resulting in non-significant differences in testosterone response between AEL and constant-load RT.

#### Perceptual Variables

Although there was considerable variance in the rating of perceived exertion (95% PI − 1.07 to 4.52), our point estimate suggested that AEL resulted in a higher rating of perceived exertion compared to constant-load RT (SMD = 1.72; *p* = 0.01). The elevated perceived exertion associated with AEL is unsurprising, given the increased eccentric load. Moreover, we found that three studies implemented a reduced concentric load and/or training volume in the AEL condition compared to constant-load RT, yet still reported higher ratings of perceived exertion for AEL [58, 65, 73]. This suggests that merely adjusting training load and volume has limited effectiveness in alleviating perceived exertion in AEL.

Our pooled analyses showed that AEL elicited similar muscle soreness both immediately after the intervention and during the subsequent period compared to constant-load RT (immediate: SMD = 0.28; *p* = 0.30; delayed: SMD = 0.18; *p* = 0.28). This finding aligns with our aforementioned results, which demonstrated that AEL and constant-load RT produced comparable muscle damage responses—one of the primary contributors to muscle soreness [98]. Notably, among the three studies in this review that examined the impact of AEL on delayed muscle soreness, one reported significantly higher delayed muscle soreness in the AEL condition compared to constant-load RT [73], differing from the other two studies [22, 90]. This discrepancy is likely attributed to differences in training design. Specifically, Merrigan and Jones [90] and Tseng et al. [22] employed three sets of a single exercise with a 3-min inter-set rest period, whereas Vincent et al. [73] used two sets of six exercises with only a 1-min inter-set rest period. This latter design is more likely to cause fatigue accumulation and insufficient recovery in AEL due to the additional eccentric load, which could lead to higher delayed muscle soreness. Thus, to prevent excessive muscle soreness when implementing AEL, it is recommended that excessively short inter-set rest periods (e.g., ≤ 1 min) and high training volumes are avoided.

### Chronic Adaptations

#### Strength-Related Adaptations

Our findings indicated that AEL yielded similar maximal strength adaptations to those of constant-load RT, including maximal dynamic concentric strength (SMD = 0.12; *p* = 0.41), maximal dynamic eccentric strength (SMD = 0.19; *p* = 0.58), and maximal voluntary isometric force (SMD = 0.03; *p* = 0.93). In principle, the primary distinction between AEL and constant-load RT is that AEL utilizes a greater eccentric load, which theoretically should result in greater adaptations in maximal dynamic eccentric strength. However, this expected outcome was not observed. In this review, two studies examined the chronic effects of AEL on maximal dynamic eccentric strength [72, 99]. Both favored AEL over constant-load RT, but neither reached statistical significance. One study suggested that the relatively short intervention duration (4 weeks, eight sessions) might have masked the true differences between AEL and constant-load RT [99]. Similarly, the other study implemented only a 5-week intervention, which is insufficient to fully understand the long-term effects of AEL on maximal dynamic eccentric strength [72]. To gain deeper insights into the chronic effects of AEL on maximal dynamic eccentric strength, future research should consider extending the intervention period.

It is worth noting that two included studies reported significantly greater improvements in maximal dynamic concentric strength with AEL compared to constant-load RT [29, 71]. One of these studies, conducted by Abougamil [29], involved professional weightlifters and utilized a high concentric and eccentric load combined with substantial training volume. Specifically, their participants were required to train three times per week, performing two to seven sets of five exercises per session with concentric loads of 80–90% 1RM and eccentric loads of 90–120% 1RM. This intensive training protocol likely enabled the AEL group to accumulate significant training stimuli from the additional eccentric load, leading to a better adaptation in maximal dynamic concentric strength. However, such a demanding regimen may not be suitable for the general population and is better recommended for individuals who typically require high training stimuli to achieve meaningful training adaptations, such as elite weightlifters and powerlifters. Another study by Brandenburg and Docherty [71] examined the chronic effects of AEL on maximal dynamic concentric strength in elbow flexor and extensor exercises. The results showed that AEL produced a better adaptation for the elbow extensors but not for the elbow flexors. The authors attributed this difference to variations in intrinsic muscle fiber structure. The elbow extensors are pennate muscles designed for high force production, which may adapt more effectively to the additional eccentric load compared to the parallel-fiber structure of the elbow flexors [100, 101]. However, few studies have directly compared the effects of AEL across different exercises. This hypothesis warrants further investigation in future research.

Our findings indicated that AEL resulted in similar adaptations in countermovement jump height (SMD = 0.04; *p* = 0.87) compared to constant-load RT. In this review, two included studies separately examined the chronic effects of AEL on countermovement jump height using either weight releasers [72] or manual weight adjustments [59]. Both studies consistently found that although AEL could improve countermovement jump height, the additional eccentric load in AEL did not provide any added training benefits compared to constant-load RT. Given the lack of differences in strength and structural adaptations between AEL and constant-load RT, and noting that the higher loads associated with AEL may alter movement/technique and increase acute perceived effort, we recommend prioritizing constant-load RT for developing long-term strength-related adaptations.

#### Structural Adaptations

Our pooled analysis demonstrated that AEL and constant-load RT led to similar muscle architectural adaptations, including fascicle angle (SMD =  − 0.10; *p* = 0.77), fascicle length (SMD = 0.90; *p* = 0.17), and muscle cross-sectional area (SMD =  − 0.06; *p* = 0.84), while there was considerable variability in muscle cross-sectional area (95% PI − 3.54 to 3.42). In this review, three studies examined the chronic effects of AEL on muscle cross-sectional area [28, 63, 71]. Among them, two studies reported non-significant increases in muscle cross-sectional area following AEL interventions [63, 71]. The first study, conducted by Brandenburg and Docherty [71], implemented a 9-week training program focusing on elbow flexor and extensor exercises. The results showed no improvements in muscle cross-sectional area for either AEL or constant-load RT. The authors attributed this to their method of cross-sectional area measurement, which assessed the maximal muscle cross-sectional area perpendicular to the length of the humerus. They suggested that hypertrophy of the elbow extensors might not have been detectable with this approach, as increases in myofibrillar content could occur along the pennate orientation, which this method did not capture. Another study by Friedmann et al. [63] also reported non-significant improvements in muscle cross-sectional area in both AEL and constant-load RT. However, this study was substantially underpowered to detect adaptations and implemented only a 4-week intervention, which was likely too short to elicit measurable changes in muscle hypertrophy [102, 103].

### Risk of Bias

Based on our risk of bias assessment, the AEL literature still has considerable room for improvement in the transparency of randomization and allocation procedures. Many studies only broadly stated that a “randomized design” was used, but did not provide sufficient information to judge how the random sequence was generated (e.g., computer-generated random numbers, block randomization, or Latin-square/counterbalancing for crossover designs) or whether allocation concealment was implemented. Future studies should clearly report the randomization and allocation process in the methods section to reduce selection bias and order effects, and to enhance reproducibility and credibility. In addition, AEL studies rarely reported whether blinding was applied to outcome assessors, making detection bias difficult to judge. We recommend reporting blinding outcome assessors whenever possible. Finally, many studies did not indicate whether they were preregistered, resulting in reporting bias being judged as “unclear” in those cases. Future research should prioritize trial registration and/or publicly available protocols and provide a statement of consistency with the registration information at publication, thereby reducing selective reporting and facilitating the accumulation and synthesis of AEL evidence.

Beyond study design and reporting, AEL interventions are particularly susceptible to implementation-related sources of bias that may confound interpretation if not controlled or clearly described. First, the effective eccentric-overload “dose” should be operationalized and reported explicitly (e.g., AEL applied on every repetition vs. only the first repetition), because these approaches represent meaningfully different stimuli and fatigue–potentiation profiles [26, 86, 90]. Second, different AEL delivery modes (e.g., weight releasers, motor-driven systems, pneumatic/electromagnetic devices, manual load adjustments) can introduce protocol-dependent procedural differences that affect reproducibility and the accuracy of the intended stimulus [19–22]. For example, weight-releaser or manual-loading protocols may require assistants to reattach/disengage loads between repetitions, introducing brief inter-repetition pauses. If unstandardized or unreported, these “micro-rests” effectively act as rest redistribution and may influence velocity, electromyography, and metabolic responses beyond the eccentric overload itself. Accordingly, researchers should report sufficient technical and procedural details (e.g., device settings, loading/reattachment procedures, repetition-to-repetition timing, and any pauses introduced) to support reproducibility and to verify that the intended eccentric overload was delivered as planned. Finally, if an AEL intervention incorporates additional training-structure variables, the control condition should be designed to match these features, or an additional matched control group should be included. For example, AEL may be combined with a rest-redistribution configuration, whereas the control condition uses constant-load RT with a traditional set structure [86]. In this case, differences in set structure may act as a co-intervention by altering recovery kinetics and thereby influencing outcomes. Therefore, including a matched control group is necessary to isolate the effects of AEL.

### Publication Bias

In our analyses, funnel plots and Egger’s tests suggested potential small-study effects (and possible publication bias) for concentric mechanical performance at submaximal loads (*p* = 0.02) and electromyography during the eccentric phase (*p* = 0.02). In both cases, the scatter was largely concentrated in the lower (less precise) region of the funnel plots, indicating that higher-precision/larger studies were scarce. Accordingly, the corresponding pooled estimates should be interpreted cautiously, as they may be prone to effect-size inflation. In addition, publication bias was assessed only for outcomes with ten or more available studies; therefore, only a small subset of variables could be evaluated (acute variables: concentric mechanical performance at submaximal loads, blood lactate, electromyography during the concentric phase, and electromyography during the eccentric phase; chronic variable: concentric 1RM), and the presence of publication bias for most other outcomes remains unknown.

### Limitations

Several limitations should be acknowledged when interpreting the findings of this review. First, although the inclusion criteria did not restrict participants’ age or training experience, the majority of participants were young, trained individuals. This may limit the generalizability of our findings to other populations, such as older adults or other vulnerable groups. Second, certain confounding factors may have influenced our findings. For example, concentric mechanical performance at submaximal loads can be affected by training variables such as the external loads and within-set rest strategies (e.g., rest-redistribution). In addition, both the number of AEL repetitions per set and the method used to implement AEL may act as potential confounders. For instance, applying AEL to every repetition versus the first repetition only likely produces different training stimuli; likewise, manual assistance versus computer-driven devices may differ in movement continuity, potentially affecting acute responses and chronic adaptations. However, given the study’s scope and the limited number of studies for most outcomes, further subgroup analyses and meta-regressions to identify these moderators could not be performed. Third, while subgroup analyses were conducted to explore differences among various AEL conditions, the limited number of included studies restricted these analyses to specific outcomes. For example, subgroup analysis of the immediate response of creatine kinase could not be performed due to the insufficient number of included studies. Consequently, we were unable to provide a comprehensive understanding of how different AEL protocols influence acute responses and chronic adaptations. Finally, some outcomes were associated with high levels of heterogeneity. Although subgroup analyses were performed, the sources of this heterogeneity could not be fully determined. We speculate that the small number of included studies is a significant contributor to the observed heterogeneity.

## Conclusion

The current systematic review and meta-analysis compared the effects of AEL and constant-load RT on acute mechanical, metabolic, and perceptual responses, as well as chronic strength-related and muscle architectural adaptations. Our findings suggest that AEL can be utilized to acutely increase athletes’ muscle electrical activity during the eccentric phase as an alternative to constant-load RT methods. However, AEL does not enhance concentric muscle electrical activity or concentric mechanical performance at submaximal loads. Furthermore, additional eccentric loads in AEL do not enhance gains in strength-related or muscle architectural adaptations compared to constant-load RT. It is important for researchers, sports professionals, and athletes to note that despite AEL not appearing to induce greater muscle damage, muscle soreness, or post-exercise mechanical fatigue, there are higher perceptions of exertion and increased metabolic stress. Therefore, incorporating longer and more frequent inter-set rest periods, as well as extended recovery between sessions, should be considered when implementing AEL [104].

## Supplementary Information

Below is the link to the electronic supplementary material.

Supplementary file1 (DOCX 14 KB)

Supplementary file2 (XLSX 14 KB)

Supplementary file3 (DOCX 76 KB)

Supplementary file4 (DOCX 292 KB)

Supplementary file5 (DOCX 23 KB)

## Declarations

### Availability of data and material

The datasets generated during and/or analyzed during the current review are available from the corresponding author on reasonable request.
