# Supplementary material for: Acute and Chronic Effects of Accentuated Eccentric Loading vs. Constant-Load Resistance Training: A Systematic Review and Meta-analysis
Source: Sports Med. 2026 Apr 7;56(7):1749–70. doi: 10.1007/s40279-026-02422-7 (PMC13388742; doi:10.1007/s40279-026-02422-7)
Supplement: Supplementary file 1 — Supplementary file1 (DOCX 14 KB) [file 40279_2026_2422_MOESM1_ESM.docx]

**Supplementary 1**

**Table 1** The specific search strategies for each database.

| **Database** | **Search strategy** | **Date** |
| --- | --- | --- |
| Web of science | (((((TS=(Accentuated eccentric load*)) OR TS=(Eccentric overload*)) OR TS=(Additional Eccentric load*)) OR TS=(Enhanced Eccentric load*)) OR TS=(Eccentric focused training)) OR TS=(Augmented eccentric load*) | 07/01 |
| PubMed | (((((Accentuated eccentric load*[Title/Abstract]) OR (Eccentric overload*[Title/Abstract])) OR (Additional Eccentric load*[Title/Abstract])) OR (Enhanced Eccentric load*[Title/Abstract])) OR (Eccentric focused training[Title/Abstract])) OR (Augmented eccentric load*[Title/Abstract]) | 07/02 |
| EBSCO | AB Accentuated eccentric load* OR AB Eccentric overload* OR AB Additional Eccentric load* OR AB Enhanced Eccentric load* OR AB Eccentric focused training OR AB Augmented eccentric load* | 07/03 |
| Embase | 'accentuated eccentric load*':ti,ab,kw OR 'eccentric overload*':ti,ab,kw OR 'additional eccentric load*':ti,ab,kw OR 'enhanced eccentric load*':ti,ab,kw OR 'eccentric focused training':ti,ab,kw OR 'augmented eccentric load*':ti,ab,kw | 07/03 |
