# Supplementary material for: Acute and Chronic Effects of Accentuated Eccentric Loading vs. Constant-Load Resistance Training: A Systematic Review and Meta-analysis
Source: Sports Med. 2026 Apr 7;56(7):1749–70. doi: 10.1007/s40279-026-02422-7 (PMC13388742; doi:10.1007/s40279-026-02422-7)
Supplement: Supplementary file 3 — Supplementary file3 (DOCX 76 KB) [file 40279_2026_2422_MOESM3_ESM.docx]

**Supplementary 3**

**Table 1** The summary of the studies included in the review.

| **Study** | **Participants’ information** | |  | **Intervention design** | | | | **Outcome** |
| --- | --- | --- | --- | --- | --- | --- | --- | --- |
|  | **Sample size (sex)** | **Age**  **RT experience** |  | **Sets × repetitions**  **Load** | **Exercise** | **AEL method** | **Intervention**  **type** |  |
| Abougamil [1] | 10 (Unclear) | Age: ≈ 16.2 years  Weightlifters of El-saha El-shabia Club |  | CL: 2 to 7×1 to 3  80% to 90% 1RM  AEL: 2 to 7×1 to 3  80% to 90% 1RM/90% to 120% 1RM | Squat  Cheat curl  Overhead squat  Snatch pull  Snatch  Bench press  Leg press  Front squat  Power Clean  Half squat  Bicep curl  Power jerk |  | Chronic  10 weeks | 1RM |
| Armstrong et al. [2] | 9 (M) | Age: 24±2 years  RT: at least 1 year |  | CL: 1×3  60% 1RM  AEL: 1×3  60% 1RM  /110%, 120%, 130%, 140%, or 150% 1RM | Squat | Electrical motor | Acute |  |
| Balshaw et al. [3] | 10 (M) | Age: 22.2±1.3 years  RT: at least 1 year |  | CL: 3×3  85% 3RM  AEL: 3×3  85% 3RM  /120% 3RM | Knee extension | A computer-driven device | Acute | MVIF  Electromyography |
| Barstow et al. [4] | 24 (Unclear) | Age: ≈ 22.2 years  RT: at least 3 months |  | CL: 3×6 to 10  60% 1RM  AEL: 3×6 to 10  60% 1RM  /100% 1RM | Arm curl | Mechanical device | Chronic  12 weeks | 1RM |
| Bartolomei et al. [5] | 11 (M) | Age: 25.6±3.9 years  RT: 6.3±3.4 years |  | CL: 6×5  80% 1RM  AEL: 6×5  80% 1RM  /120% 1RM | Bench press | Manual | Acute | Acute muscle swelling |
| Brandenburg and Docherty [6] | 18 (M) | Age: university-aged  RT: at least 1 year |  | CL: 4×10  75% 1RM  AEL: 3×10  75% 1RM  /120% 1RM | Elbow flexors  Elbow extensors | Manual | Chronic  9 weeks | 1RM  Muscle cross-section area |
| Castro et al. [7] | 12 (M) | Age: 26±6 years  RT: at least 1 year |  | CL: 2×1  30% or 80% 1RM  AEL: 2×1  30% or 80% 1RM  /100% 1RM | Bench press | Weight releasers | Acute | Electromyography  Mean velocity |
| Chae et al. [8] | 12 (M) | Age: 25.6±4.4 years  RT: at least 1 year |  | CL: 3×10  60% 1RM  AEL: 3×10  60% 1RM/110% 1RM | Squat | Weight releasers | Acute | Countermovement jump  Heart rate  Lactate  Rating of perceived exertion |
| Chae et al. [9] | 12 (M) | Age: 25.6±4.4 years  RT: at least 1 year |  | CL: 3×10  60% 1RM  AEL: 3×10  60% 1RM/110% 1RM | Squat | Weight releasers | Acute | Mean velocity |
| Doan et al. [10] | 8 (M) | Age: 23.9 years  RT: experienced |  | CL: unclear×1  100% 1RM  AEL: unclear×1  100% 1RM/105% 1RM | Bench press | Weight releasers | Acute | 1RM |
| Douglas et al. [11] | 14 (M) | Age: 19.4±0.8 years  RT: at least 1 year |  | CL: 2 to 5×4 to 8  74% to 85% 1RM  AEL: 2 to 5×4 to 8  68% to 81% 1RM/92% to 110% 1RM | Squat | Pneumatic assistance | Chronic  8 weeks | 1RM  Fascicle length  Fascicle angle |
| English et al. [12] | 16 (M) | Age: 34.9±7.0 years  RT: at least 0.5 years untrained |  | CL: 2 to 5×2 to 8  55% to 96% 1RM  AEL: 2 to 5×2 to 8  55% to 96% 1RM/CON+38% | Leg press  Calf press | Electrical motor | Chronic  8 weeks | 1RM |
| Friedmann-Bette et al. [13] | 25 (M) | Age: ≈ 24.4 years  RT: average 5 years |  | CL: 6×8  8RM  AEL: 5×8  8RM/190% 8RM | Knee extension | A computer-driven device | Chronic  6 weeks |  |
| Friedmann et al. [14] | 16 (M) | Age: ≈ 24.5 years  Untrained |  | CL: 6×25  30% 1RM  AEL: 3×25  30% 1RM/70% 1RM | Knee extension | A computer-driven device | Chronic  4 weeks | Muscle cross-section area |
| Godard et al. [15] | 18  (Unclear) | Age: ≈ 21.6 years  Untrained |  | CL: 1×8 to 12  80% 1RM  AEL: 1×8 to 12  80% 1RM/CON+40% | Knee extension | A computer-driven device | Chronic  10 weeks | 1RM |
| Harden et al. [16] | 12 (M) | Age: 31±6 years  RT: 12±9 years |  | CL: 1×1  1RM  AEL: 1×1  50% 1RM/100% ECC1RM | Leg press | Pneumatic assistance | Acute |  |
| Harden et al. [17] | 12  (9M+3F) | Age: ≈ 27 years  RT: 3 to 10 years |  | CL: 4×3  82.5% to 102.5% 1RM  AEL: 4×3  82.5 to 102.5% 1RM/82.5% to 102.5% ECC1RM | Leg press | Pneumatic assistance | Chronic  4 weeks | 1RM |
| Kaminski et al. [18] | 18 (M) | Age: 22.9±3.1 years  RT: at least 0.5 years untrained |  | CL: 2×8  80% 1RM  AEL: 2×8  40% 1RM/100% 1RM | Leg curl | Mechanical device | Chronic  6 weeks |  |
| Kristiansen et al. [19] | 10 (M) | Age: 24.0±6.4 years  RT: at least 0.5 years |  | CL: 1×2  85% 1RM  AEL: 1×2  85% 1RM/110% 1RM | Bench press | Weight releasers | Acute | Electromyography  Peak velocity |
| Lanza et al. [20] | 15 (F) | Age: 21±2 years  RT: at least 0.5 years untrained |  | CL: 3×8  60% 1RM  AEL: 3×8  60% 1RM/84% 1RM | Leg curl | Mechanical device | Acute | Electromyography |
| Lates et al. [21] | 13 (M) | Age: 23.7±4.0 years  RT: 7.2±2.4 years |  | CL: unclear×5  80% 1RM  AEL: unclear×5  80% 1RM/105% 1RM | Bench press | Weight releasers | Acute | Mean velocity |
| Liu et al. [22] | 14 (M) | Age: unclear  Chinese provincial team athletes |  | CL: 1 to 2×7  80% 1RM  AEL: 1 to 2×7  50% 1RM/80% 1RM | Half squat | Weight releasers | Acute | Countermovement jump |
| Merrigan and Jones [23] | 21 (M) | Age: 24.0±4.2 years  RT: 6.8±3.0 years |  | CL: 3×3 to 5  65% or 80% 1RM  AEL: 3×3 to 5  65% or 80% 1RM/120% 1RM | Squat | Weight releasers | Acute | Acute muscle swelling  Cortisol  Soreness |
| Merrigan et al. [24] | 21 (M) | Age: 24.0±4.2 years  RT: 6.8±3.0 years |  | CL: 3×3 to 5  65% or 80% 1RM  AEL: 3×3 to 5  65% or 80% 1RM/120% 1RM | Squat | Weight releasers | Acute | Mean velocity |
| Merrigan et al. [25] | 10  (8M+2F) | Age: ≈ 25 years  RT: at least 1 year |  | CL: 4×5  50% or 65% 1RM  AEL: 4×5  50% or 65% 1RM/120% 1RM | Bench press | Weight releasers | Acute | Mean velocity |
| Moore et al. [26] | 13 (M) | Age: 22.8±2.9 years  RT: at least 0.5 years |  | CL: 2×1  30% 1RM  AEL: 2×1  30% 1RM/CON+20%, 50%, or 80% | Squat jump | Weight releasers | Acute | Peak velocity |
| Munger et al. [27] | 23 (M) | Age: 23.4±2.7 years  RT: at least 1 year |  | CL: 3 to 4×2 to 5  80% to 90% 1RM  AEL: 3 to 4×2 to 5  55% to 65% 1RM/105% to 115% 1RM | Squat | Weight releasers | Chronic  5 weeks | Countermovement jump  1RM |
| Ojasto and Häkkinen [28] | 11 (M) | Age: 32.4±4.3 years  RT: experienced |  | CL: 4×10  70% 1RM  AEL: 4×10  70% 1RM/80%, 90%, or 100% 1RM | Bench press | Weight releasers | Acute | Growth hormone  Lactate |
| Ojasto and Häkkinen [29] | 11 (M) | Age: 32.4±4.3 years  RT: experienced |  | CL: 1 to 2×1 to 2  50% or 100% 1RM  AEL: 1 to 2×1 to 2  50% or 100% 1RM/60%, 70%, 80%, 90%, 105%, 110%, or 120% 1RM | Bench press | Weight releasers | Acute | 1RM  Peak power  Electromyography |
| Raeder et al. [30] | 15 (M) | Age: 23.1±1.9 years  RT: at least 1 year |  | CL: 4×6  85% 1RM  AEL: 4×6  70% 1RM/100% 1RM | Squat | Manual | Acute | Countermovement jump  MVIF  Creatine kinase  Lactate  Rating of perceived exertion |
| Sarto et al. [31] | 10 (M) | Age: 23.3±2.1 years  RT: at least 3 years |  | CL: 2×6  70% and 80% 1RM  AEL: 2×6  70% and 80% 1RM/CON+50% | Leg press | Electrical motor | Acute | Electromyography |
| Sheppard and Young [32] | 14 (M) | Age: 25.0±1.0 years  Members of a national sports training academy |  | CL: 2×1  40 kg  AEL: 2×1  40 kg/60, 70, and 80 kg | Bench throw | Height-adjustable clamp | Acute |  |
| Simola et al. [33] | 14 (M) | Age: 23.0±1.9 years  RT: at least 2 years |  | CL: 4×6  85% 1RM  AEL: 4×6  70% 1RM/100% 1RM | Squat | Manual | Acute | MVIF  Lactate  Rating of perceived exertion |
| Suchomel et al. [34] | 16 (M) | Age: 24.4±3.8 years  RT: at least 1 year |  | CL: 4×3  50% to 80% 1RM  AEL: 4×3  50% to 80% 1RM/100% or 110% 1RM | Squat | Weight releasers | Acute | Mean velocity |
| Taber et al. [35] | 10 (M) | Age: 23±3 years  RT: at least 1 year |  | CL: 6×1  30% to 80% 1RM  AEL: 6×1  30% to 80% 1RM/100% or 110% 1RM | Bench press | Weight releasers | Acute |  |
| Taber et al. [36] | 21  (16M+5F) | Age: 23.5±1.8 years  RT: 6.4 ± 3.2 year |  | CL: 4×2  20 to 50 kg  AEL: 4×2  20 to 50 kg/CON+10 kg, 20 kg, or 30 kg | Barbell loaded countermovement jump  Trap bar loaded countermovement jump | Weight releasers | Acute |  |
| Toien et al. [37] | 33 (M) | Age: 23±3 years  Untrained |  | CL: 4×4  90% to 95% 1RM  AEL: 4×4  90% to 95% 1RM/150%1RM | Leg press | Manual | Chronic  8 weeks | 1RM  Countermovement jump |
| Tseng et al. [38] | 16 (M) | Age: 18 to 25 years  RT: at least 2 years |  | CL: 3×5  85% 1RM  AEL: 3×4  80% 1RM/105% 1RM | Half squat | Manual | Acute | Countermovement jump  Soreness |
| van den Tillaar and Kwan [39] | 16 (M) | Age: 28.5±7.7 years  RT: at least 1 year |  | CL: 1×3  85% 1RM  AEL: 1×3  85% 1RM/95% 1RM | Bench press | Weight releasers | Acute | Electromyography  Peak velocity |
| Vincent et al. [40] | 20  (14M+6F) | Age: 26.8±5.9 years  RT: at least 1 year |  | CL: 2×12  60% 1RM  AEL: 2×10  50% 1RM/100% 1RM | Leg extension  Leg curl  Chest press  Seated row  Shoulder press  Pull-down | Electrical motor | Acute | Heart rate  Creatine kinase  Lactate  Rating of perceived exertion  Soreness |
| Wagle et al. [41] | 11 (M) | Age: 26.1±4.1 years  RT: at least 1 year |  | CL: 3×5  80% 1RM  AEL: 3×5  80% 1RM/105% 1RM | Squat | Weight releasers | Acute | Peak power |
| Wagle et al. [42] | 11 (M) | Age: 26.1±4.1 years  RT: at least 1 year |  | CL: 3×5  80% 1RM  AEL: 3×5  80% 1RM/105% 1RM | Squat | Weight releasers | Acute |  |
| Walker et al. [43] | 20 (M) | Age: 21±3 years  RT: 2.6±2.2 years |  | CL: 3×failure  6RM and 10RM  AEL: 3×failure  6RM and 10RM/CON+40% | Bilateral leg press  Unilateral knee extension  Bilateral knee flexion | Weight releasers | Chronic  10 weeks | Fascicle length  Fascicle angle  MVIF |
| Walker et al. [44] | 20 (M) | Age: 22±3 years  RT: 2.6±2.2 years |  | CL: 3×failure  6RM and 10RM  AEL: 3×failure  6RM and 10RM/CON+40% | Bilateral leg press  Unilateral knee extension  Unilateral knee flexion | Weight releasers | Chronic  10 weeks | 1RM  Muscle cross-section area |
| Walker et al. [45] | 17 (M) | Age: ≈ 21 years  RT: 2.7±2.3 years |  | CL: 3×failure  6RM and 10RM  AEL: 3×failure  6RM and 10RM/CON+40% | 45° leg press  Unilateral knee extension | Weight releasers  Manual | Chronic  10 weeks | Growth hormone  Cortisol  Testosterone  Lactate  MVIF |
| Wehrstein et al. [46] | 30 (M) | Age: ≈ 24 years  An average of three workouts per week |  | CL: 6×8  80% 1RM  AEL: 6×8  60°·s^-1^/CON+30% | Leg extension | A computer-driven device | Acute | Creatine kinase |
| Yarrow et al. [47] | 22 (M) | Age: 22.1±0.8 years  Untrained |  | CL: 4×6  52.5% to 75% 1RM  AEL: 3×6  40% to 49% 1RM/  100% to 121% 1RM | Bench press  Squat | Electrical motor | Chronic  5 weeks | Growth hormone  Testosterone  Lactate  Rating of perceived exertion  1RM |
| Yarrow et al. [48] | 22 (M) | Age: 21.9±0.8 years  Untrained |  | CL: 4×6  52.5% 1RM  AEL: 3×6  40% 1RM  /100% 1RM | Bench press  Squat | Electrical motor | Acute | Growth hormone  Testosterone  Lactate  Rating of perceived exertion |
| Zambrano et al. [49] | 16  (10M+6F) | Age: 25.8±5.5 years  RT: 6.7±4.4 years |  | CL: 1×failure  10RM  AEL: 1×failure  10RM/CON+50% | Elbow flexion | Electromagnetic resistance | Acute | Electromyography |

**Note**: F, female; M, male; RT, resistance training; CL, constant-load; AEL, accentuated eccentric loading (training load in accentuated eccentric loading was presented as concentric load/eccentric load, such as 50% 1RM/100% 1RM); MVIF, maximal voluntary isometric force; 1RM, one repetition maximal; CON, concentric; ECC, eccentric.

**References**

1. Abougamil E. The effect of a training program using concentric/overload eccentric exercises on maximum strength, achievement record and some dynamic variables of snatch lift. Turkish Journal of Kinesiology. 2017;3(4):77-85.

2. Armstrong R, Baltzopoulos V, Langan-Evans C, Clark D, Jarvis J, Stewart C, et al. An investigation of movement dynamics and muscle activity during traditional and accentuated-eccentric squatting. PLoS ONE. 2022;17(11):1-19.

3. Balshaw TG, Pahar M, Chesham R, Macgregor LJ, Hunter AM. Reduced firing rates of high threshold motor units in response to eccentric overload. Physiological reports. 2017 Jan;5(2).

4. Barstow IK, Bishop MD, Kaminski TW. Is enhanced-eccentric resistance training superior to traditional training for increasing elbow flexor strength? Journal of sports science & medicine. 2003;2(2):62.

5. Bartolomei S, Totti V, Nigro F, Ciacci S, Semprini G, Di Michele R, et al. A Comparison Between The Recovery Responses Following an Eccentrically Loaded Bench Press Protocol Vs. Regular Loading in Highly Trained Men. Journal of Human Kinetics. 2019 Aug;68(1):59-67.

6. Brandenburg JP, Docherty D. The effects of accentuated eccentric loading on strength, muscle hypertrophy, and neural adaptations in trained individuals. Journal of Strength and Conditioning Research. 2002;16(1):25-32.

7. Castro AH, Zangakis D, Moir GL. The Effects of Accentuated Eccentric Loading on Mechanical Variables and Agonist Electromyography during the Bench Press. Sports. 2020 Jun;8(6).

8. Chae S, Long SA, Lis RP, McDowell KW, Wagle JP, Carroll KM, et al. Combined Accentuated Eccentric Loading and Rest Redistribution in High-Volume Back Squat: Acute Stimulus and Fatigue. Journal of strength and conditioning research. 2024;38(4):648-55.

9. Chae S, Long SA, Lis RP, McDowell KW, Wagle JP, Carroll KM, et al. Combined Accentuated Eccentric Loading and Rest Redistribution in High-Volume Back Squat: Acute Kinetics and Kinematics. Journal of strength and conditioning research. 2024;38(4):640-7.

10. Doan BK, Newton RU, Marsit JL, Triplett-McBride N, Koziris LP, Fry AC, et al. Effects of increased eccentric loading on bench press 1RM. Journal of Strength and Conditioning Research. 2002 Feb;16(1):9-13.

11. Douglas J, Pearson S, Ross A, McGuigan M. Effects of Accentuated Eccentric Loading on Muscle Properties, Strength, Power, and Speed in Resistance-Trained Rugby Players. Journal of strength and conditioning research. 2018;32(10):2750-61.

12. English KL, Loehr JA, Lee SMC, Smith SM. Early-phase musculoskeletal adaptations to different levels of eccentric resistance after 8 weeks of lower body training. European Journal of Applied Physiology. 2014 Nov;114(11):2263-80.

13. Friedmann-Bette B, Bauer T, Kinscherf R, Vorwald S, Klute K, Bischoff D, et al. Effects of strength training with eccentric overload on muscle adaptation in male athletes. European Journal of Applied Physiology. 2010;108(4):821-36.

14. Friedmann B, Kinscherf R, Vorwald S, Müller H, Kucera K, Borisch S, et al. Muscular adaptations to computer-guided strength training with eccentric overload. Acta physiologica Scandinavica. 2004;182(1):77-88.

15. Godard MP, Wygand JW, Carpinelli RN, Catalano S, Otto RM. Effects of accentuated eccentric resistance training on concentric knee extensor strength. Journal of Strength and Conditioning Research. 1998 Feb;12(1):26-9.

16. Harden M, Wolf A, Haff GG, Hicks KM, Howatson G. Repeatability and Specificity of Eccentric Force Output and the Implications for Eccentric Training Load Prescription. Journal of Strength and Conditioning Research. 2019 Mar;33(3):676-83.

17. Harden M, Wolf A, Evans M, Hicks KM, Thomas K, Howatson G. Four weeks of augmented eccentric loading using a novel leg press device improved leg strength in well-trained athletes and professional sprint track cyclists. Plos One. 2020 Jul;15(7).

18. Kaminski TW, Wabbersen CV, Murphy RM. Concentric versus enhanced eccentric hamstring strength training: Clinical implications. Journal of Athletic Training. 1998 Jul-Sep;33(3):216-21.

19. Kristiansen EL, Larsen S, van den Tillaar R. The Acute Effect of Accentuated Eccentric Overloading upon the Kinematics and Myoelectric Activity in the Eccentric and Concentric Phase of a Traditional Bench Press. Sports (Basel, Switzerland). 2021 Dec 29;10(1).

20. Lanza MB, Pertence AEM, Andrade AGP, Peixoto GH, Bonato Felix L, Chagas MH. Acute neuromuscular response during eccentric overload protocol by using a mechanical device to increase the load. Brazilian Journal of Kineanthropometry & Human Performance. 2017;19(4):375-84.

21. Lates AD, Greer BK, Wagle JP, Taber CB. Accentuated Eccentric Loading and Cluster Set Configurations in the Bench Press. Journal of Strength and Conditioning Research. 2022 Jun;36(6):1485-9.

22. Liu Y, Zhang J, Hu Z, Zhong Z, Yuan X. Acute effects of eccentric overload training with different loading doses in male sprinters. Heliyon. 2024 Jun 15;10(11):e32369.

23. Merrigan JJ, Jones MT. Acute Inflammatory, Cortisol, and Soreness Responses to Supramaximal Accentuated Eccentric Loading. Journal of strength and conditioning research. 2021;35:S107-S13.

24. Merrigan JJ, Tufano JJ, Falzone M, Jones MT. Effectiveness of Accentuated Eccentric Loading: Contingent on Concentric Load. International Journal of Sports Physiology and Performance. 2021 Jan;16(1):66-72.

25. Merrigan JJ, Tufano JJ, Jones MT. Potentiating Effects of Accentuated Eccentric Loading Are Dependent Upon Relative Strength. Journal of Strength and Conditioning Research. 2021 May;35(5):1208-16.

26. Moore CA, Weiss LW, Schilling BK, Fry AC, Li YH. Acute effects of augmented eccentric loading on jump squat performance. Journal of Strength and Conditioning Research. 2007 May;21(2):372-7.

27. Munger CN, Jones BC, Halloran IJ, Eggleston GG, Post PG, Brown LE, et al. Short-term effects of eccentric overload versus traditional back squat training on strength and power. International Journal of Kinesiology and Sports Science. 2022;10(1):1-8.

28. Ojasto T, Häkkinen K. Effects of different accentuated eccentric loads on acute neuromuscular, growth hormone, and blood lactate responses during a hypertrophic protocol. Journal of Strength and Conditioning Research. 2009 May;23(3):946-53.

29. Ojasto T, Häkkinen K. Effects of different accentuated eccentric load levels in eccentric-concentric actions on acute neuromuscular, maximal force, and power responses. Journal of Strength and Conditioning Research. 2009 May;23(3):996-1004.

30. Raeder C, Wiewelhove T, Westphal-Martinez MP, Fernandez-Fernandez J, de Paula Simola RA, Kellmann M, et al. Neuromuscular Fatigue and Physiological Responses After Five Dynamic Squat Exercise Protocols. Journal of Strength and Conditioning Research. 2016 Apr;30(4):953-65.

31. Sarto F, Franchi MV, Rigon PA, Grigoletto D, Zoffoli L, Zanuso S, et al. Muscle activation during leg-press exercise with or without eccentric overload. European Journal of Applied Physiology. 2020 Jul;120(7):1651-6.

32. Sheppard JM, Young K. Using additional eccentric loads to increase concentric performance in the bench throw. Journal of Strength and Conditioning Research. 2010 Oct;24(10):2853-6.

33. Simola RAD, Harms N, Raeder C, Kellmann M, Meyer T, Pfeiffer M, et al. ASSESSMENT OF NEUROMUSCULAR FUNCTION AFTER DIFFERENT STRENGTH TRAINING PROTOCOLS USING TENSIOMYOGRAPHY. Journal of Strength and Conditioning Research. 2015 May;29(5):1339-48.

34. Suchomel TJ, Cantwell CJ, Campbell BA, Schroeder ZS, Marshall LK, Taber CB. Braking and Propulsion Phase Characteristics of Traditional and Accentuated Eccentric Loaded Back Squats. Journal of Human Kinetics. 2024 Mar;91(1):121-33.

35. Taber CB, Morris JR, Wagle JP, Merrigan JJ. Accentuated Eccentric Loading in the Bench Press: Considerations for Eccentric and Concentric Loading. Sports. 2021 May;9(5).

36. Taber C, Butler C, Dabek V, Kochan B, McCormick K, Petro E, et al. The effects of accentuated eccentric loading on barbell and trap bar countermovement jumps. International Journal of Strength and Conditioning. 2023;3(1).

37. Toien T, Haglo HP, Unhjem R, Hoff T, Wang E. Maximal strength training: the impact of eccentric overload. Journal of Neurophysiology. 2018 Dec;120(6):2868-76.

38. Tseng KW, Chen JR, Chow JJ, Tseng WC, Condello G, Tai HL, et al. Post-activation Performance Enhancement after a Bout of Accentuated Eccentric Loading in Collegiate Male Volleyball Players. International Journal of Environmental Research and Public Health. 2021 Dec;18(24).

39. van den Tillaar R, Kwan K. The Effects of Augmented Eccentric Loading upon Kinematics and Muscle Activation in Bench Press Performance. J Funct Morphol Kinesiol. 2020 Jan 26;5(1).

40. Vincent HK, Percival S, Creasy R, Alexis D, Seay A, Ann ZL, et al. Acute effects of enhanced eccentric and concentric resistance exercise on metabolism and inflammation. Journal of novel physiotherapies. 2014;4(2).

41. Wagle JP, Cunanan AJ, Carroll KM, Sams ML, Wetmore A, Bingham GE, et al. Accentuated Eccentric Loading and Cluster Set Configurations in the Back Squat: A Kinetic and Kinematic Analysis. Journal of Strength and Conditioning Research. 2021 Feb;35(2):420-7.

42. Wagle JP, Taber CB, Carroll KM, Cunanan AJ, Sams ML, Wetmore A, et al. Repetition-to-Repetition Differences Using Cluster and Accentuated Eccentric Loading in the Back Squat. Sports (Basel, Switzerland). 2018 Jul 8;6(3).

43. Walker S, Trezise J, Haff GG, Newton RU, Häkkinen K, Blazevich AJ. Increased fascicle length but not patellar tendon stiffness after accentuated eccentric-load strength training in already-trained men. European Journal of Applied Physiology. 2020 Nov;120(11):2371-82.

44. Walker S, Blazevich AJ, Haff G, Tufano JJ, Newton RU, Häkkinen K. Greater Strength Gains after Training with Accentuated Eccentric than Traditional Isoinertial Loads in Already Strength-Trained Men. Frontiers in Physiology. 2016 Apr;7.

45. Walker S, Häkkinen K, Haff GG, Blazevich AJ, Newton RU. Acute elevations in serum hormones are attenuated after chronic training with traditional isoinertial but not accentuated eccentric loads in strength-trained men. Physiological reports. 2017 Apr;5(7).

46. Wehrstein M, Schöffel A, Weiberg N, Gwechenberger T, Betz T, Rittweg M, et al. Eccentric Overload during Resistance Exercise: A Stimulus for Enhanced Satellite Cell Activation. Medicine & Science in Sports & Exercise. 2022 Mar;54(3):388-98.

47. Yarrow JF, Borsa PA, Borst SE, Sitren HS, Stevens BR, White LJ. Early-Phase Neuroendocrine Responses and Strength Adaptations Following Eccentric-Enhanced Resistance Training. Journal of Strength and Conditioning Research. 2008 Jul;22(4):1205-14.

48. Yarrow JF, Borsa PA, Borst SE, Sitren HS, Stevens BR, White LJ. Neuroendocrine responses to an acute bout of eccentric-enhanced resistance exercise. Medicine & Science in Sports & Exercise. 2007;39(6):941-7.

49. Zambrano H, Torres X, Coleman M, Franchi MV, Fisher JP, Oberlin D, et al. Myoelectric activity during electromagnetic resistance alone and in combination with variable resistance or eccentric overload. Scientific Reports. 2023 May 22;13(1).
