# Supplementary material for: Acute and Chronic Effects of Accentuated Eccentric Loading vs. Constant-Load Resistance Training: A Systematic Review and Meta-analysis
Source: Sports Med. 2026 Apr 7;56(7):1749–70. doi: 10.1007/s40279-026-02422-7 (PMC13388742; doi:10.1007/s40279-026-02422-7)
Supplement: Supplementary file 4 — Supplementary file4 (DOCX 292 KB) [file 40279_2026_2422_MOESM4_ESM.docx]

**Supplementary 4**

**Acute variables**


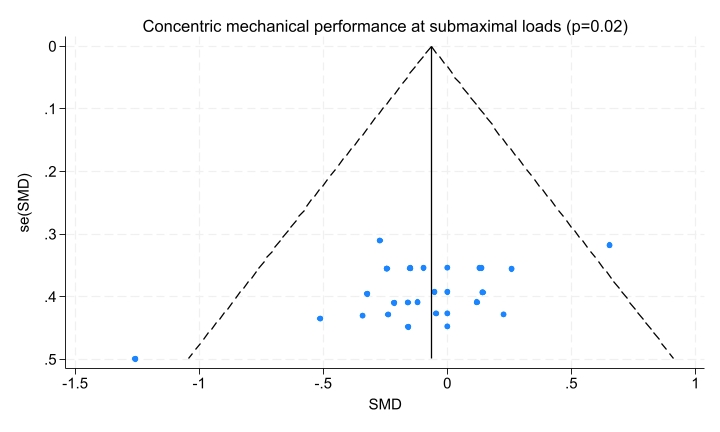


**Figure 1** The funnel plot and Egger's test of concentric mechanical performance at submaximal loads (during).


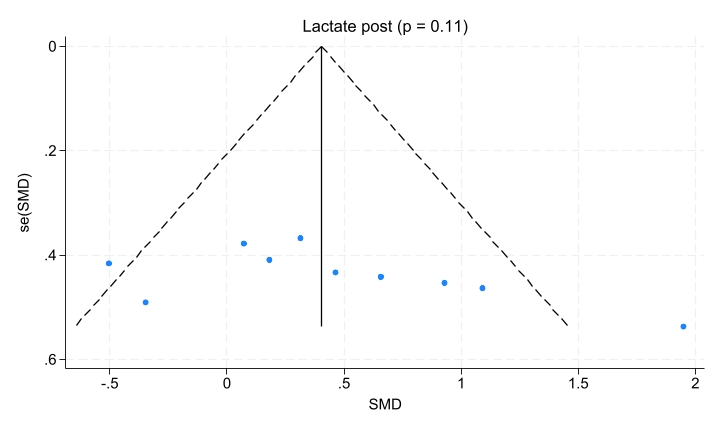


**Figure 2** The funnel plot and Egger's test of blood lactate (immediate).


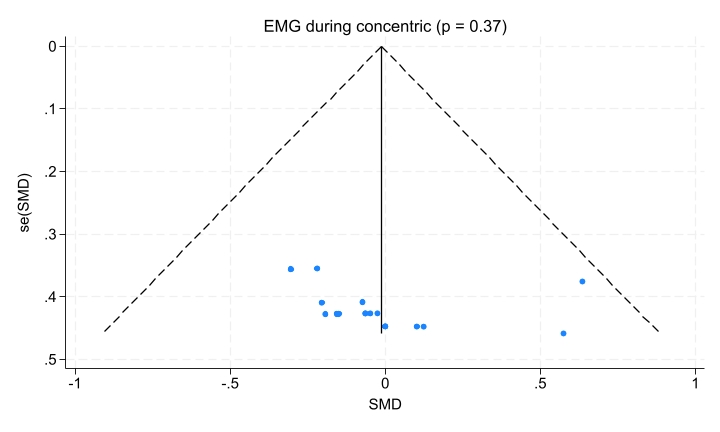


**Figure 3** The funnel plot and Egger's test of concentric electromyography (during).


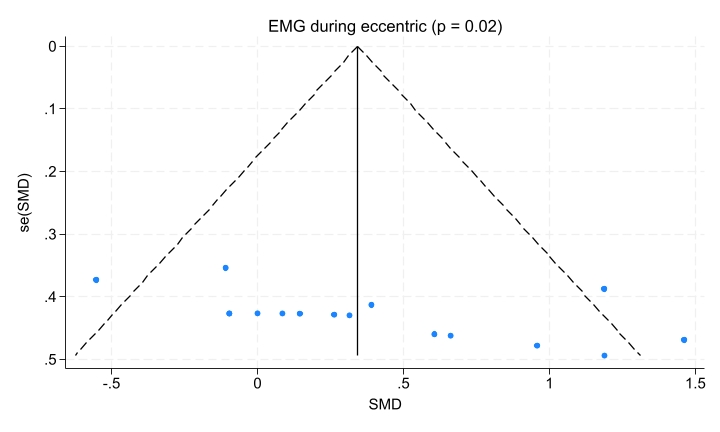


**Figure 4** The funnel plot and Egger's test of eccentric electromyography (during).

**Chronic variable**


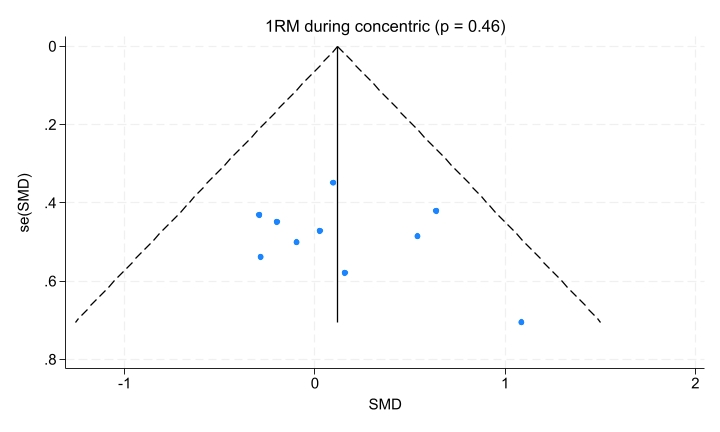


**Figure 5** The funnel plot and Egger's test of concentric 1RM.
