# Supplementary material for: Acute and Chronic Effects of Accentuated Eccentric Loading vs. Constant-Load Resistance Training: A Systematic Review and Meta-analysis
Source: Sports Med. 2026 Apr 7;56(7):1749–70. doi: 10.1007/s40279-026-02422-7 (PMC13388742; doi:10.1007/s40279-026-02422-7)
Supplement: Supplementary file 5 — Supplementary file5 (DOCX 23 KB) [file 40279_2026_2422_MOESM5_ESM.docx]

**Supplementary 5**

**Table 1** The results of sensitivity analysis.

| **Outcome** |  | | **Meta-analysis** | | | | |
| --- | --- | --- | --- | --- | --- | --- | --- |
|  | **k** | **SMD** | | **95%CI** | **95%PI** | ***p*** | ***I*^2^** |
| **Concentric 1RM (During)** |  |  | |  |  |  |  |
| Accentuated eccentric loading | 4 | -0.16 | | -0.59 to 0.28 | -1.11 to 0.80 | 0.48 | 0% |
| Supramaximal subgroup | 4 | -0.16 | | -0.59 to 0.28 | -1.11 to 0.80 | 0.48 | 0% |
| **CMPSL (During)** |  |  | |  |  |  |  |
| Accentuated eccentric loading | 29 | -0.07 | | -0.21 to 0.08 | -0.21 to 0.08 | 0.37 | 0% |
| Submaximal subgroup | 8 | -0.03 | | -0.31 to 0.25 | -0.38 to 0.32 | 0.84 | 0% |
| Maximal subgroup | 6 | -0.13 | | -0.42 to 0.17 | -0.55 to 0.29 | 0.41 | 0% |
| Supramaximal subgroup | 15 | -0.06 | | -0.26 to 0.14 | -0.33 to 0.21 | 0.57 | 4% |
| **Lactate (Immediate)** |  |  | |  |  |  |  |
| Accentuated eccentric loading | 6 | 0.30 | | -0.22 to 0.81 | -1.19 to 1.78 | 0.26 | 53% |
| Submaximal subgroup | 2 | 0.76 | | 0.14 to 1.38 | k < 3 | 0.02 | 0% |
| Maximal subgroup | 1 | - | | - | - | - | - |
| Supramaximal subgroup | 3 | -0.21 | | -0.70 to 0.29 | -3.39 to 2.98 | 0.41 | 0% |
| **Growth hormone (Immediate)** |  |  | |  |  |  |  |
| Accentuated eccentric loading | 4 | 0.50 | | 0.06 to 0.94 | -0.47 to 1.46 | 0.03 | 0% |
| Submaximal subgroup | 2 | 0.53 | | -0.14 to 1.20 | k < 3 | 0.12 | 18% |
| Maximal subgroup | 1 | - | | - | - | - | - |
| Supramaximal subgroup | 1 | - | | - | - | - | - |
| **Creatine kinase (Immediate)** |  |  | |  |  |  |  |
| Accentuated eccentric loading | 2 | 0.09 | | -0.38 to 0.55 | k < 3 | 0.72 | 0% |
| **Acute muscle swelling (Immediate)** |  |  | |  |  |  |  |
| Accentuated eccentric loading | 3 | 0.26 | | -0.37 to 0.88 | -6.46 to 6.98 | 0.42 | 59% |
| Supramaximal subgroup | 3 | 0.26 | | -0.37 to 0.88 | -6.46 to 6.98 | 0.42 | 59% |
| **Concentric electromyography (During)** |  |  | |  |  |  |  |
| Accentuated eccentric loading | 17 | -0.01 | | -0.21 to 0.18 | -0.23 to 0.20 | 0.90 | 0% |
| Submaximal subgroup | 6 | 0.01 | | -0.31 to 0.33 | -0.45 to 0.46 | 0.96 | 0% |
| Maximal subgroup | 2 | -0.14 | | -0.71 to 0.43 | k < 3 | 0.63 | 0% |
| Supramaximal subgroup | 9 | 0.00 | | -0.28 to 0.28 | -0.34 to 0.34 | 0.99 | 0% |
| **Eccentric electromyography (During)** |  |  | |  |  |  |  |
| Accentuated eccentric loading | 16 | 0.37 | | 0.09 to 0.65 | -0.49 to 1.23 | 0.01 | 44% |
| Submaximal subgroup | 6 | 0.24 | | -0.24 to 0.72 | -1.16 to 1.63 | 0.33 | 53% |
| Maximal subgroup | 2 | 0.90 | | -0.15 to 1.95 | k < 3 | 0.09 | 66% |
| Supramaximal subgroup | 8 | 0.33 | | -0.02 to 0.69 | -0.47 to 1.14 | 0.07 | 28% |
| **Concentric 1RM** |  |  | |  |  |  |  |
| Accentuated eccentric loading | 7 | 0.18 | | -0.17 to 0.52 | -0.27 to 0.63 | 0.31 | 0% |
| Maximal subgroup | 1 | - | | - | - | - | - |
| Supramaximal subgroup | 5 | 0.07 | | -0.33 to 0.47 | -0.58 to 0.73 | 0.73 | 0% |
| **MVIF** |  |  | |  |  |  |  |
| Accentuated eccentric loading | 2 | 0.03 | | -0.62 to 0.67 | k < 3 | 0.93 | 0% |
| Supramaximal subgroup | 2 | 0.03 | | -0.62 to 0.67 | k < 3 | 0.93 | 0% |

**Note:** k, number of trials; SMD, standardized mean differences (a positive SMD indicates higher values for accentuated eccentric loading, while a negative SMD indicates higher values for constant-load resistance training); CMPSL, concentric mechanical performance at submaximal loads; CI, confidence intervals; PI, prediction intervals, MVIF, maximal voluntary isometric force.
